# Development of a venous thromboembolism risk prediction model for patients with primary membranous nephropathy based on machine learning

**DOI:** 10.3389/fphar.2025.1683708

**Published:** 2025-11-06

**Authors:** Lian Li, Liuyun Wu, Yin Wang, Hulin Wang, Xingyue Zheng, Lizhu Han, Qinan Yin, Xingwei Wu, Yuan Bian

**Affiliations:** 1 Department of Pharmacy, Personalized Drug Research and Therapy Key Laboratory of Sichuan Province, Sichuan Provincial People’s Hospital, School of Medicine, University of Electronic Science and Technology of China, Chengdu, China; 2 The Fourth People’s Hospital of Chengdu, Chengdu, China; 3 Department of Pharmacy, Chengdu Women’s and Children’s Central Hospital, School of Medicine, University of Electronic Science and Technology of China, Chengdu, China

**Keywords:** primary membranous nephropathy, venous thromboembolism, riskfactors, machine learning, prediction model

## Abstract

**Objective:**

This study utilizes real-world data from primary membranous nephropathy (PMN) patients to preliminarily develop a venous thromboembolism (VTE) risk prediction model with machine learning. The aim is to improve the rational use of prophylactic anticoagulant therapy by predicting VTE risk in these patients.

**Methods:**

We collected diagnostic and treatment data for PMN patients hospitalized at Sichuan Provincial People’s Hospital from 1 January 2018, to 30 September 2024. The data was divided into training and test sets at an 8:2 ratio, followed by processed using combinations of three imputation methods, three sampling methods, and three feature selection methods. After preprocessing, fourteen machine learning algorithms were employed to develop a predictive model for VTE risk in PMN patients. The SHapley Additive exPlanation (SHAP) method was used to interpret the contribution of outcome features. Finally, a VTE risk prediction tool for PMN patients was constructed using Streamlit.

**Results:**

A total of 643 patients with PMN were included in the study, of whom 93 developed VTE. Among the 504 models constructed, the NGBoost model, which incorporated imputation by K-Nearest Neighbor, sampling by Borderline-SMOTE, and feature selection by Frequency-based Selection, was identified as the optimal model, achieving an area under the curve (AUC) of 0.911. The optimal model included ten features: D-dimer (DD), Fibrin Degradation Products (FDP)>5 mg/L, international normalized ratio (INR) of prothrombin, Recurrent nephrotic syndrome (RNS), cholinesterase (CHE), Urinary Microalbumin to Creatinine Ratio (umALB/Ucr), statins, antithrombin III (AT III) activity, albumin, and anti-phospholipase A2 receptor antibody (aPLA2Rab). Finally, an online predictive tool based on the optimal model was developed to provide real-time individualized VTE risk predictions for PMN patients.

**Conclusion:**

This study developed a personalized risk prediction model for VTE in PMN patients using machine learning techniques. Additionally, a web-based tool for this predictive model was created. The model demonstrates strong predictive performance and can assist in clinical decision-making for the prevention and treatment of VTE in PMN patients.

## 1 Introduction

Membranous Nephropathy (MN) is primarily characterized by proteinuria and hypoalbuminemia, typically progressing to Nephrotic Syndrome (NS). MN can be classified into primary membranous nephropathy (PMN) and secondary membranous nephropathy (SMN) based on etiology, with PMN accounting for 80% of cases (Couser, 2017; Ronco and Debiec, 2015; Dc and Pe, 2017). In China, the proportion of PMN among primary glomerulonephritis cases has risen from 9.89% (1979–2002) to 18.42% (2003–2014), indicating a yearly increase in incidence (Hladunewich et al., 2009; Hou et al., 2018).

It is currently believed that PMN patients experience prolonged heavy proteinuria and hypoalbuminemia, which result in the loss of small-molecule proteins. In response, the liver compensates by synthesizing large-molecule proteins, causing abnormalities such as platelet activation, abnormal coagulation system test indexes, and reduced fibrinolytic system activity. These abnormalities contribute to a hypercoagulable state in PMN patients, making them highly susceptible to VTE (Lionaki et al., 2012; Bierzynska and Saleem, 2017; Cheung et al., 2018).

VTE is one of the most common complications of PMN, encompassing deep vein thrombosis (DVT) and pulmonary embolism (PE), as well as intra-abdominal venous thrombosis (Li et al., 2012). Among these, renal vein thrombosis (RVT) is the most prevalent, with reported incidences varying widely between 29% and 60% across different studies (Velasquez Forero et al., 1988; Singhal and Brimble, 2006; Li et al., 2012). Additionally, the occurrence of VTE is associated with the severity of proteinuria and hypoalbuminemia in PMN patients and typically manifests early in the disease course (Lionaki et al., 2012; Vestergaard et al., 2022). During the progression of PMN, the risk of VTE rapidly increases from less than 1% at the disease onset to 7%, particularly when serum albumin (ALB) levels are ≤28 g/L. Below this threshold, for every 10 g/L decrease in serum ALB, the risk of thromboembolic events nearly doubles (Lionaki et al., 2012; Couser, 2017). Research shows a markedly elevated risk of thromboembolism early in the PMN disease course, especially in the nephrotic state; the VTE incidence was 9.85% within the first 6 months (Mahmoodi et al., 2008).

However, the current predictive indicators for assessing VTE risk in PMN patients are primarily based on serum ALB levels. Recent studies have identified associations between VTE occurrence in PMN patients and factors such as D-dimer levels, proteinuria, anti-phospholipase A2 receptor antibody (aPLA2Rab) levels, and low-density lipoprotein cholesterol (LDL-C) levels (Kumar et al., 2012; Li et al., 2012; Wu et al., 2022; Zhu et al., 2022). Consequently, a scientifically rigorous method for assessing VTE risk in PMN patients remains elusive, and there is a lack of effective predictive models for the risk of VTE formation in these patients. As a core technology of artificial intelligence, machine learning is often employed to construct data analysis and predictive models. This study aims to incorporate multiple types of factors and use machine learning algorithms to develop a VTE risk prediction model for PMN patients. This model could provide crucial support for devising individualized prophylactic anticoagulation treatment strategies for PMN patients.

## 2 Materials and methods

### 2.1 Data explanation

The data for this study were obtained from the electronic health record system (EHRS) of Sichuan Provincial People’s Hospital and through telephone follow-up. The study included PMN patients who were hospitalized at Sichuan Provincial People’s Hospital between 1 January 2018, and 30 September 2024. For patients with multiple hospitalization records, in order to achieve the goal of early prediction of VTE risk and to avoid the confounding factors introduced by changes and interventions during multiple treatment processes, this study extracts data records from the patients’ first admission, and in cases where the same indicator is recorded multiple times, the initial record is taken. Inclusion criteria required patients to be diagnosed with MN via renal biopsy or to have a positive aPLA2Rab test. Exclusion criteria were as follows: (1) SMN, including hepatitis B- and C-associated MN, systemic lupus erythematosus-associated MN, thyroiditis-associated MN, tumor-associated MN, Sjögren’s syndrome-associated MN, HIV-associated MN, and ankylosing spondylitis-associated MN; (2) hospitalization due to VTE; (3) diagnosed with VTE within 24 h of hospitalization; (4) death during hospitalization; (5) inability to obtain diagnostic and treatment data; (6) lack of contact information.

The outcome variable of this study is the diagnosis of VTE-related diseases within 6 months of the initial hospitalization, such as VTE, DVT, RVT, and PE, as confirmed by vascular ultrasound examination.

In the course of the study, patients’ private information, such as names, addresses, and contact approaches, was anonymized. This study was conducted in accordance with the Declaration of Helsinki and was approved by the institutional ethics committee.

### 2.2 Statistical analysis

Continuous variables are expressed as mean ± standard deviation (SD). For variables following a normal distribution, the t-test is used, while the Mann-Whitney U test is employed for non-normally distributed variables. Categorical variables are presented as frequencies and percentages (%), and analyzed using the chi-squared test and Fisher’s exact test. A p-value of <0.05 is considered statistically significant. Statistical analyses are conducted using the stats module in Python 3.8, and model development is performed using the sklearn library in Python 3.8.

### 2.3 Variable processing

Variables with more than 90% missing values, those where the largest category accounts for more than 90% of records, those with more than 90% maximum category count, and those with a coefficient of variation less than 0.01 were excluded. Binary categorical variables were assigned values of 0 or 1. Ordinal categorical variables were assigned values of 1, 2, 3, and so on. Nominal categorical variables were transformed using one-hot encoding; for variables with more than five categories, consolidation was performed prior to encoding.

Three data imputation methods were employed: (1) Simple Imputation: For continuous variables, the median was used as the imputation value, while for categorical variables, the mode was used. (2) K-Nearest Neighbors (KNN) Imputation: The KNN algorithm was utilized to predict and replace the missing values. (3) Random Forest (RF) Imputation: The RF algorithm was applied to predict and replace the missing values. RF classification models are used for imputing categorical variables, while RF regression models are applied for imputing continuous variables. To prevent data leakage during imputation, all target labels are excluded, and only feature values are retained for the imputation process. After imputation, a consistent 8:2 train-test split was applied to all three datasets using a fixed random seed, ensuring identical samples in each respective set and producing three training sets (n = 514) and three test sets (n = 129).

Three data sampling methods were employed: (1) Synthetic Minority Oversampling Technique (SMOTE): synthesizes and supplements new samples from the minority class using a subset of original data. (2) Borderline-SMOTE (BSMOTE): an enhanced algorithm based on SMOTE that selectively generates new samples only from the minority class samples on the border, thereby improving the distribution of class samples. (3) Adaptive Synthetic (ADASYN): dynamically generates data based on the difficulty of learning from minority class samples. To counteract the continuous values generated during categorical variables imputation, the values were rounded to the nearest categories. The sample sampling was conducted solely on the training sets to allow the models to fully learn minority class characteristics, while the test sets retained their original imbalanced distribution for a realistic performance assessment. This step produced nine training sets.

Three feature screening methods were employed: (1) non-screening: models were built using all variables. (2) Lasso selection: a feature screening method based on linear regression, accurately identifying important variables. (3) LightGBM selection: utilizing the LightGBM algorithm to assess the importance of feature variables and select the top ten ranked features. (4) Frequency-based Selection (Freq-based): After performing feature screening with LASSO and LightGBM, the frequency of each feature’s occurrence was quantified across the 18 generated subset datasets. Subsequently, the top 10 most frequently occurring features were selected to reconstruct a refined feature selection subset. Following feature selection on the training sets, 36 sets were obtained. The test sets were then matched and supplemented accordingly based on the imputation method, retaining only the features present in their corresponding training sets.

### 2.4 Model construction

Through different data imputation techniques, data sampling, and feature screening, 36 datasets were obtained. 14 machine learning algorithms were employed, including Logistic Regression (LR), Support Vector Machine (SVM), KNN, Decision Tree, Multi-Layer Perceptron (MLP), RF, Extremely Randomized Trees (ExtRa Trees), Adaptive Boosting (AdaBoost), Gradient Boosting (GBoost), Extreme Gradient Boosting (XGBoost), Categorical Boosting (CatBoost), Natural Gradient Boosting (NGBoost), Bernoulli naive Bayes (Ber NB), and Gaussian naive Bayes (Gau NB).

The training set was used for model development and the test set was used for model evaluation. Ten-fold cross-validation was performed on the training set to internally validate the model and assess the stability of machine learning predictions across different data partitions and performance metrics.

### 2.5 Model evaluation

In this study, performance evaluation metrics of classification models were utilized, including receiver operating characteristic (ROC) curve and its AUC, precision-recall (P-R) curve, calibration curve, calibration slope and intercept, decision curve analysis (DCA), accuracy, precision, recall, F1-score, and Brier score. SHapley Additive exPlanations (SHAP) were employed to elucidate the contributions of variables to the models.

To assess the impact of different sample sizes on model prediction performance, Bootstrapping was conducted by randomly sampling subsets of 10%, 20%, 30%, up to 100% from the training set. These 10 subsets were used iteratively to build models, and their AUC was computed using a test set. This process was repeated 100 times to examine the effect of sample size. 200 bootstrap resampling iterations were performed to evaluate the impact of different methods of data preprocessing, modeling, and viable features. One-way ANOVA was applied in the feature sensitivity analysis.

### 2.6 Web-based prediction tool

Building upon the aforementioned steps, a final optimal prediction model is obtained and implemented using Streamlit to establish a web-based prediction tool. This platform incorporates multiple predictive indicators highly associated with concurrent VTE in patients with PMN, ultimately providing personalized VTE risk assessment for PMN patients.

## 3 Results

### 3.1 Research population

**FIGURE 1:**
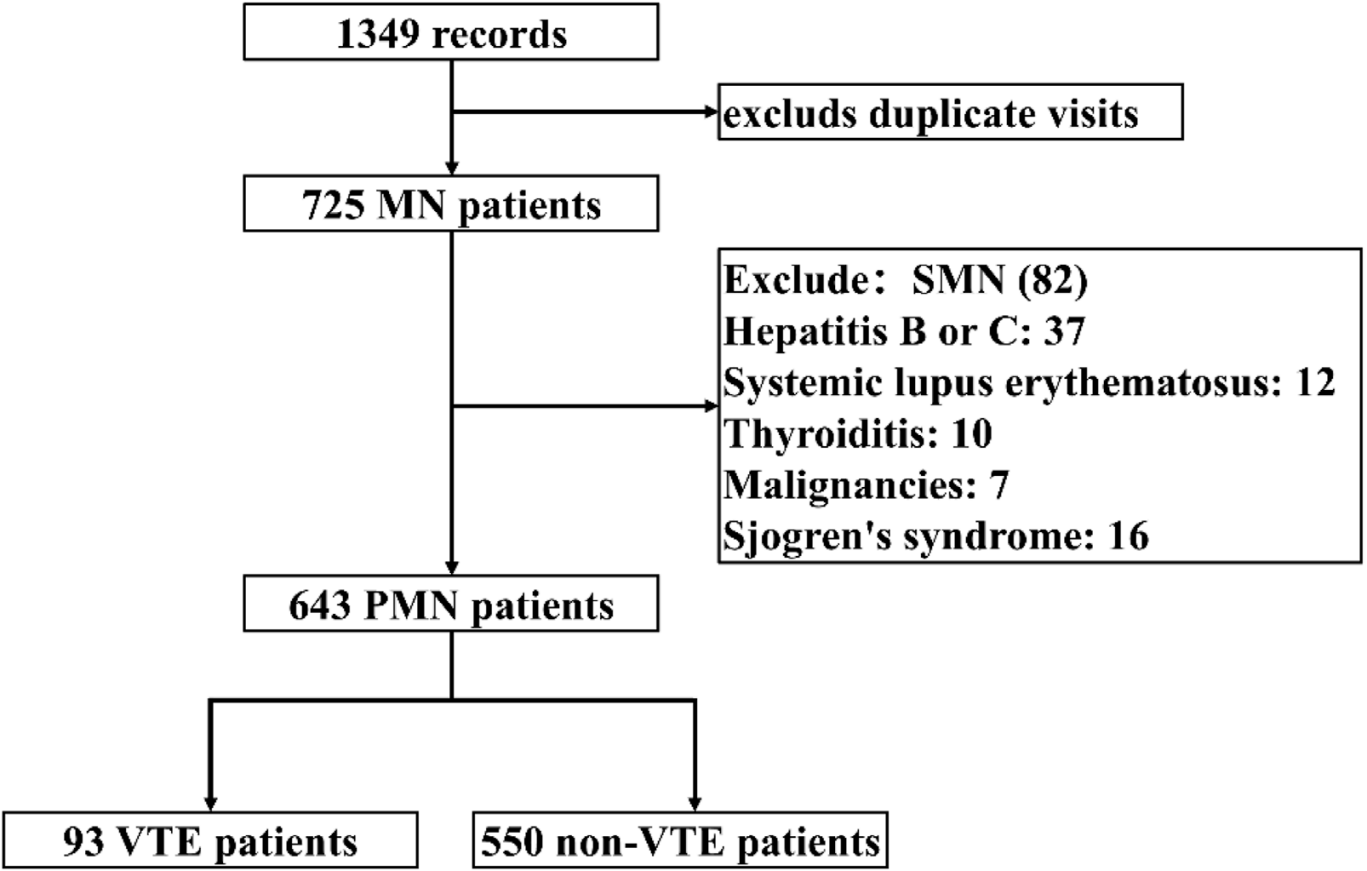
Patient inclusion flow chart.

A total of 1,349 records of hospitalized patients diagnosed with MN were extracted and 725 cases remained after excluding duplicate visits. Following inclusion and exclusion criteria screening, SMN patients were excluded, resulting in a final cohort of 643 patients. Among these, 93 cases experienced VTE, while 550 did not. Figure 1 depicts the patient enrollment process. The demographic and clinical characteristics of the patients at baseline are summarized in Supplementary Tables 1–4.

### 3.2 The results of variable procession

86 indicators were identified for inclusion after data preprocessing, being categorized into nine basic demographic features, 21 comorbidities and medical history details, five medication-related factors, and 51 laboratory test results, as detailed in Supplementary Table 6. The missing performance of each variable is presented in Supplementary Table 7. Supplementary Table 8 lists the medical reference ranges for each variable, while Supplementary Table 9 outlines the specific sub-features encompassed within the merged features, such as anticoagulants, liver diseases, etc.

The initial dataset was processed for missing values using three different imputation methods, followed by dataset splitting, resulting in 36 standardized datasets. The details for each dataset are provided in Table 1.

**TABLE 1:** Data processing combinations and dataset sizes.

Number	Fill method	Sampling method	Feature selection method	Number of variables	Training set size	Test set size
1	knn	ADA	all	62	893	129
2	knn	ADA	frequent	10	893	129
3	knn	ADA	lasso	10	893	129
4	knn	ADA	lightgbm	10	893	129
5	knn	BSMO	all	62	880	129
6	knn	BSMO	frequent	10	880	129
7	knn	BSMO	lasso	10	880	129
8	knn	BSMO	lightgbm	10	880	129
9	knn	SSMO	all	62	880	129
10	knn	SSMO	frequent	10	880	129
11	knn	SSMO	lasso	10	880	129
12	knn	SSMO	lightgbm	10	880	129
13	rf	ADA	all	62	893	129
14	rf	ADA	frequent	10	893	129
15	rf	ADA	lasso	10	893	129
16	rf	ADA	lightgbm	10	893	129
17	rf	BSMO	all	62	880	129
18	rf	BSMO	frequent	10	880	129
19	rf	BSMO	lasso	10	880	129
20	rf	BSMO	lightgbm	10	880	129
21	rf	SSMO	all	62	880	129
22	rf	SSMO	frequent	10	880	129
23	rf	SSMO	lasso	10	880	129
24	rf	SSMO	lightgbm	10	880	129
25	simple	ADA	all	62	893	129
26	simple	ADA	frequent	10	893	129
27	simple	ADA	lasso	10	893	129
28	simple	ADA	lightgbm	10	893	129
29	simple	BSMO	all	62	880	129
30	simple	BSMO	frequent	10	880	129
31	simple	BSMO	lasso	10	880	129
32	simple	BSMO	lightgbm	10	880	129
33	simple	SSMO	all	62	880	129
34	simple	SSMO	frequent	10	880	129
35	simple	SSMO	lasso	10	880	129
36	simple	SSMO	lightgbm	10	880	129

### 3.3 Model building

After fitting 36 datasets using 14 machine learning algorithms, we generated a total of 504 VTE risk prediction models for PMN patients. Preliminary assessments of model performance were conducted using 10-fold cross-validation. Selected results from the 10-fold cross-validation of some models are shown in Table 2.

**TABLE 2:** 10-fold cross-validation results of models based on training set.

Models	Algorithms	Imputation	Sampling	Selection	AUC	Accuracy	Precision	Recall	Brier	F1_Score
Optimal Model	NGBoost	KNN	BSMOTE	Freq-based	0.964 ± 0.018	0.898 ± 0.033	0.878 ± 0.048	0.927 ± 0.024	0.078 ± 0.025	0.901 ± 0.030
model_2	RF	KNN	SMOTE	LightGBM	1.000 ± 0.000	0.978 ± 0.014	0.959 ± 0.025	1.000 ± 0.000	0.022 ± 0.008	0.979 ± 0.013
model_3	NGBoost	RF	BSMOTE	ALL	0.974 ± 0.013	0.897 ± 0.039	0.871 ± 0.060	0.939 ± 0.025	0.074 ± 0.025	0.902 ± 0.033
model_4	LR	KNN	BSMOTE	freq-based	0.873 ± 0.044	0.797 ± 0.045	0.795 ± 0.053	0.805 ± 0.062	0.145 ± 0.027	0.798 ± 0.045
model_5	RF	RF	BSMOTE	LightGBM	0.987 ± 0.011	0.942 ± 0.025	0.925 ± 0.037	0.964 ± 0.025	0.052 ± 0.015	0.944 ± 0.023

NGBoost, Natural Gradient Boosting; LR, logistic regression; KNN, K-Nearest Neighbors; RF, random forest; SMOTE, synthetic minority oversampling Technique; BSMOTE, borderline-SMOTE; LightGBM, light gradient boosting machine; Freq-based, Frequency-based.

### 3.4 Model evaluation

**FIGURE 2:**
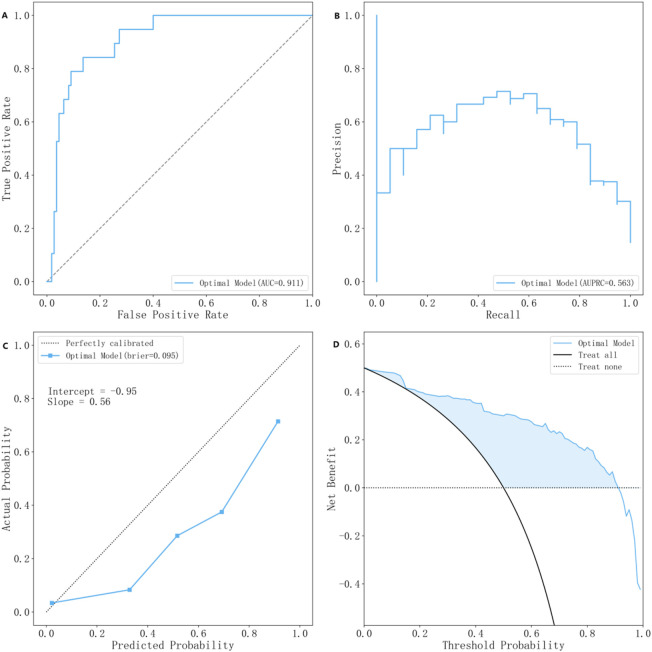
The Characteristic Curves of The Optimal Model A, the ROC curve; B, the P-R curve; C, the calibration curve; D, the clinical decision curve.

**FIGURE 3:**
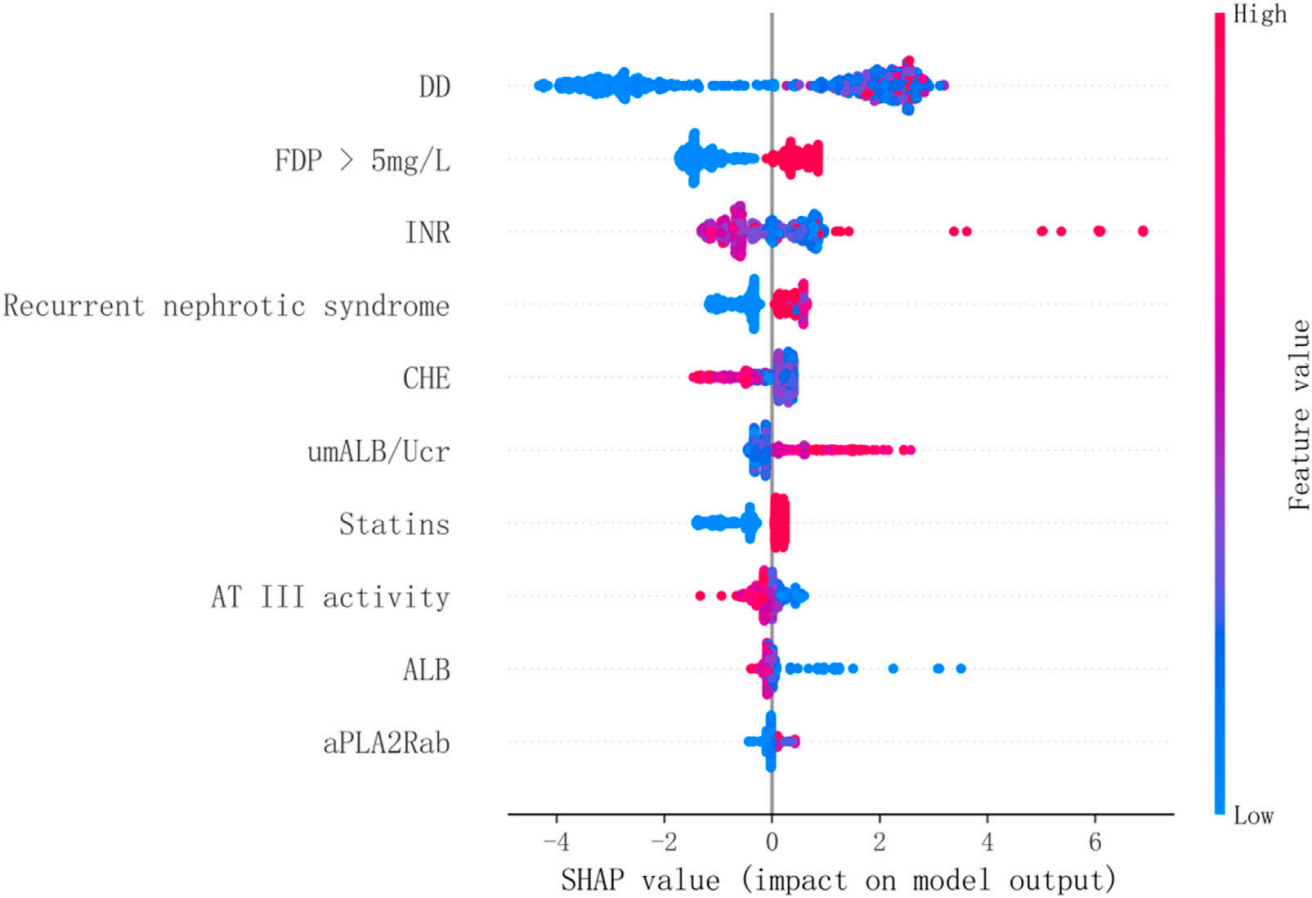
The SHAP Diagram of The Optimal Model ALB, albumin; aPLA2Rab, anti-phospholipase A2 receptor antibody; AT III, antithrombin III; CHE, cholinesterase; DD, D-dimer; INR, international normalized ratio; umALB/Ucr, Urinary Microalbumin to Creatinine Ratio; PT, prothrombin time; RNS, relapse of nephrotic syndrome Each dot represents a sample, with red indicating higher variable values and blue indicating lower variable values. For instance, as shown in the figure, elevated DD levels may be associated with an increased risk of VTE.

**FIGURE 4:**
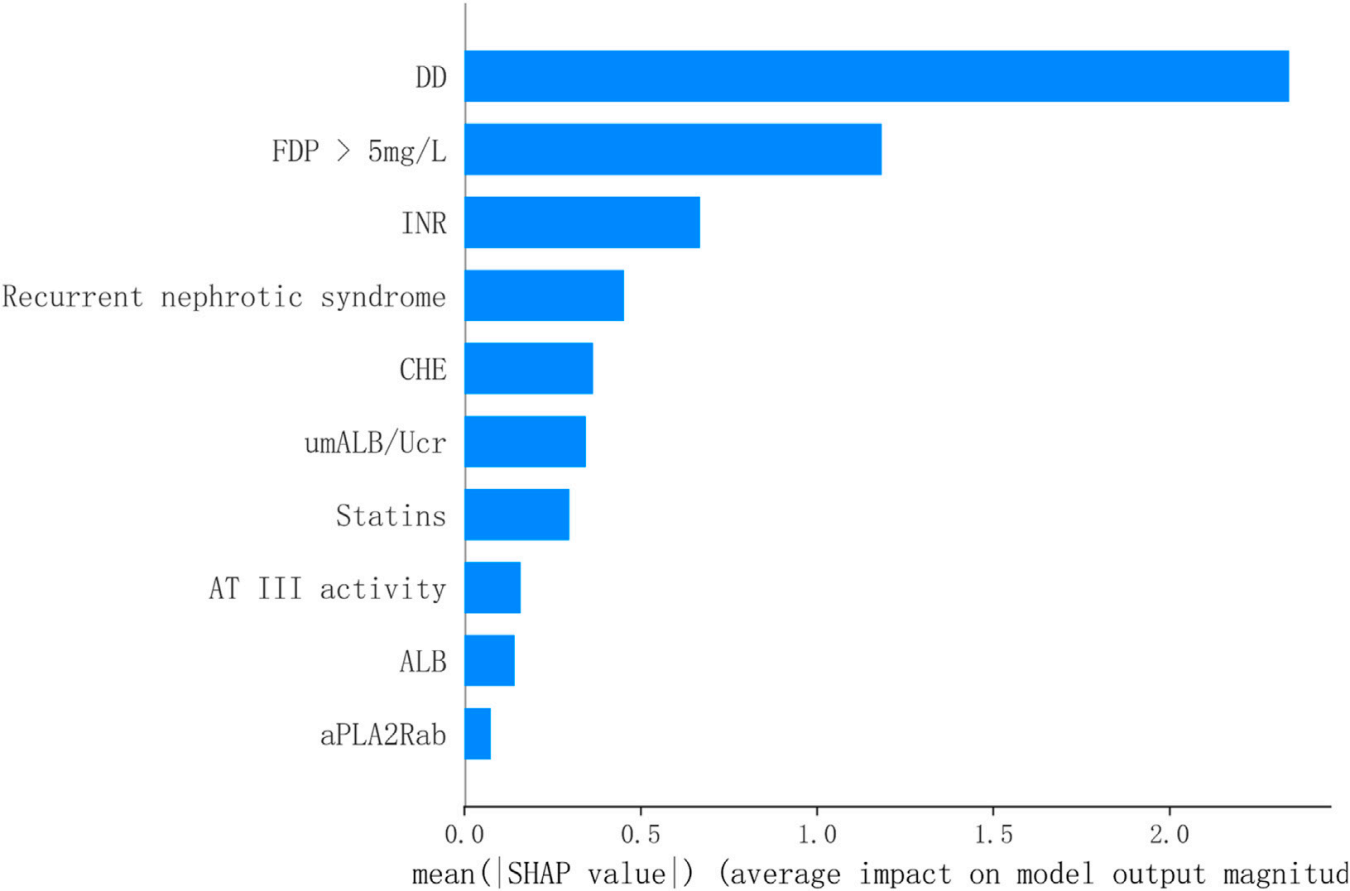
Feature importance ranking of the optimal model.

**FIGURE 5:**
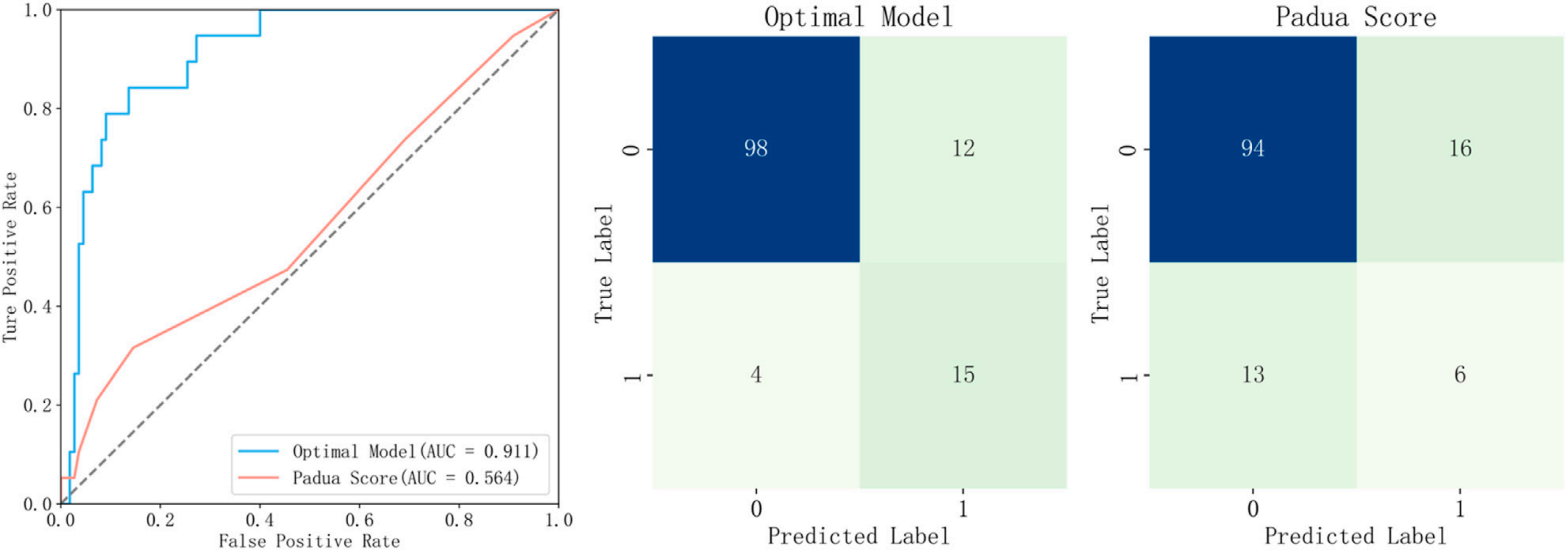
Comparison of Predictive Performance: Optimal Model vs. Padua Score.

**FIGURE 6:**
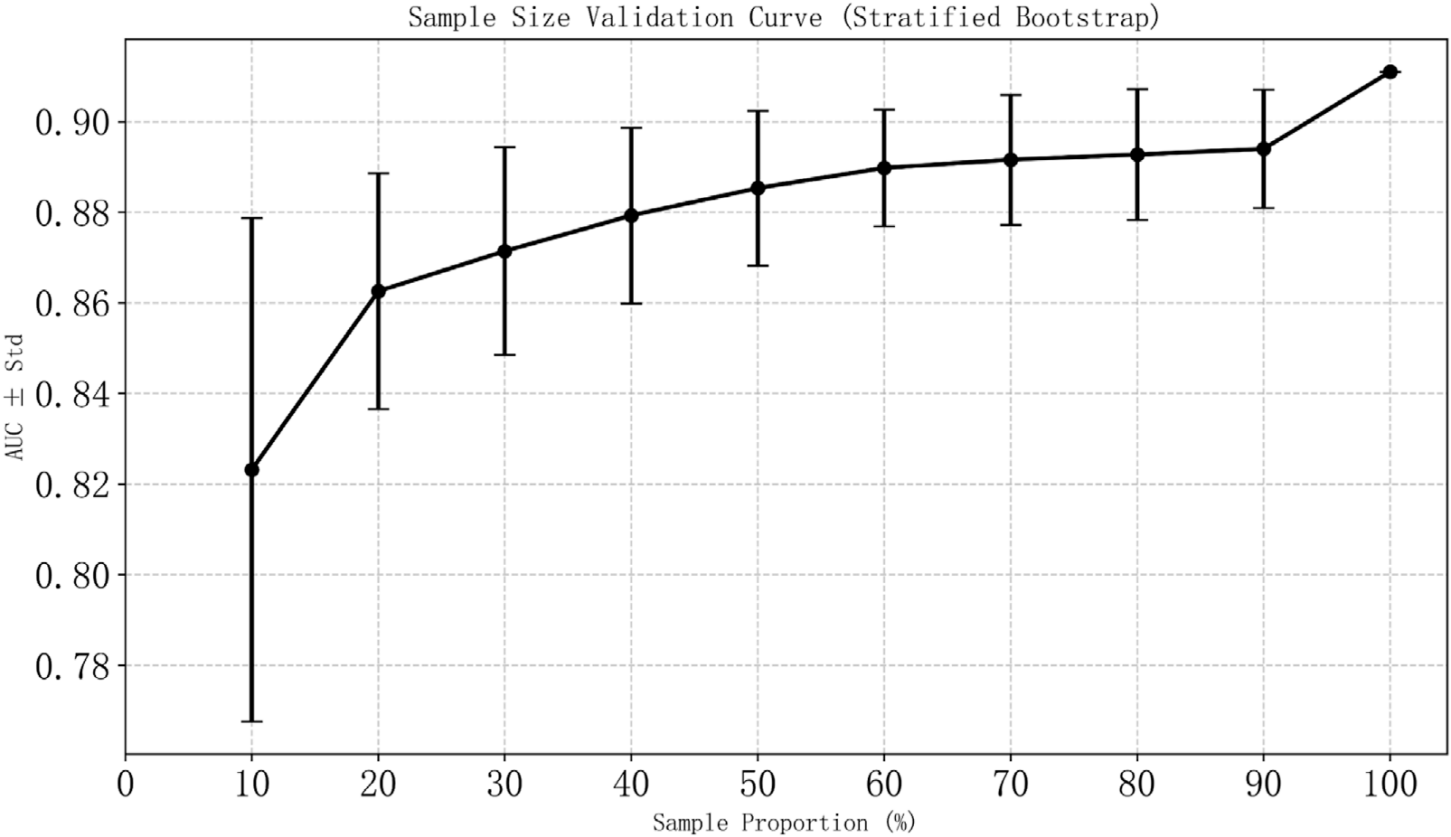
Sample size validation.

The evaluation of model performance was conducted using the test dataset, calculating metrics including AUC, accuracy, precision, recall, F1 score, and Brier score. Among the models evaluated, the NGBoost model (Optimal Model), optimized through KNN imputation, BSMO sampling, and Frequency-based feature selection, demonstrated superior comprehensive performance in both AUC (0.911) and F1-score (0.652). Optimal Model, model_2, and model_3 ranked as the top three in AUC performance, while model_4 exhibited the best performance among classical LR models, model_5 achieved the highest F1-score. Figure 2 displays the ROC curve, P-R curve, calibration curve, and decision curve for the optimal model. The model calibration evaluation resulted in a Brier score of 0.095, indicating good overall accuracy. The calibration intercept was −0.946 and the slope was 0.556, reflecting minor over-prediction for high-risk cases and under-prediction for low-risk cases. Nevertheless, the model demonstrated good discrimination and acceptable calibration. SHAP values were used to explain the contributions of variables to the models. Figure 3 illustrates the relationship between the values of each variable in the final predictive model and the corresponding changes in SHAP values. Figure 4 displays the ranking of variable importance in the final predictive model. The results indicate that D-dimer, FDP > 5 mg/L, INR, RNS, and CHE are the five most critical variables. In the predictive model, higher SHAP values for a variable suggest a greater possibility of VTE occurrence. Figure 5 compares the performance of our optimal model against the traditional Padua score using ROC curves and confusion matrices. As shown in Figure 6, the AUC value of the test set increases with the sample size, and the curve gradually flattens, demonstrating the robustness and persuasiveness of our study. A one-way ANOVA was performed based on 200 bootstrap replicates from the test set to evaluate the effects of different data preprocessing methods, algorithmic models, and features included in the optimal model. Table 3 summarizes the results. In terms of AUC, all factors except the sampling method showed statistically significant effects on model performance. The NGBoost algorithm achieved the highest AUC value. For feature sensitivity analysis, each feature was individually excluded, and the resulting model was compared against the optimal model via bootstrap resampling. As shown in Table 4, excluding DD significantly reduced the AUC from 0.896 ± 0.013 to 0.856 ± 0.016 (p < 0.01). In contrast, excluding Statins, ALB, or AT III activity led to a slight increase in AUC (p < 0.01), suggesting potential feature redundancy or noise associated with these variables in this study.

**TABLE 3:** Performance metrics of different data processing methods on model prediction performance.

Method	AUC		Accuracy		Precision		Recall		F1		Brier	
	AUC Mean ± SD	AUC 95%CI	Accuracy Mean ± SD	Accuracy 95%CI	Precision Mean ± SD	Precision 95%CI	Recall Mean ± SD	Recall 95%CI	F1 Mean ± SD	F1 95%CI	Brier Mean ± SD	Brier 95%CI
Filling Method												
knn	0.783 ± 0.010	0.764–0.802	0.797 ± 0.008	0.782–0.812	0.427 ± 0.012	0.403–0.451	0.491 ± 0.014	0.464–0.517	0.424 ± 0.010	0.406–0.443	0.156 ± 0.005	0.147–0.165
rf	0.764 ± 0.009	0.746–0.781	0.793 ± 0.008	0.777–0.809	0.427 ± 0.013	0.402–0.452	0.482 ± 0.013	0.456–0.507	0.418 ± 0.009	0.400–0.435	0.159 ± 0.005	0.149–0.169
simple	0.769 ± 0.010	0.750–0.788	0.782 ± 0.009	0.763–0.798	0.405 ± 0.013	0.379–0.431	0.473 ± 0.013	0.446–0.497	0.403 ± 0.010	0.384–0.422	0.162 ± 0.005	0.152–0.172
p value	p < 0.01		p > 0.05		p > 0.05		p > 0.05		p > 0.05		p > 0.05	
Sampling Method												
ADA	0.772 ± 0.009	0.755–0.789	0.786 ± 0.009	0.767–0.804	0.418 ± 0.011	0.396–0.441	0.506 ± 0.010	0.486–0.528	0.433 ± 0.009	0.415–0.450	0.159 ± 0.005	0.150–0.167
BSMO	0.775 ± 0.010	0.756–0.794	0.799 ± 0.007	0.784–0.813	0.426 ± 0.012	0.404–0.450	0.487 ± 0.012	0.463–0.508	0.432 ± 0.010	0.413–0.450	0.156 ± 0.005	0.147–0.165
SSMO	0.768 ± 0.010	0.748–0.788	0.786 ± 0.008	0.770–0.800	0.414 ± 0.014	0.388–0.442	0.454 ± 0.017	0.422–0.486	0.381 ± 0.010	0.361–0.401	0.164 ± 0.006	0.153–0.175
p value	p > 0.05		p > 0.05		p > 0.05		p < 0.05		p < 0.001		p > 0.05	
Feature Selection Method												
all	0.755 ± 0.012	0.731–0.776	0.772 ± 0.011	0.750–0.792	0.418 ± 0.018	0.383–0.455	0.444 ± 0.014	0.415–0.472	0.381 ± 0.010	0.360–0.403	0.176 ± 0.007	0.161–0.190
frequent	0.798 ± 0.010	0.778–0.818	0.809 ± 0.008	0.793–0.823	0.442 ± 0.013	0.416–0.468	0.536 ± 0.016	0.504–0.565	0.460 ± 0.011	0.438–0.481	0.146 ± 0.005	0.137–0.157
lasso	0.762 ± 0.010	0.742–0.782	0.784 ± 0.009	0.765–0.801	0.391 ± 0.012	0.368–0.413	0.475 ± 0.016	0.445–0.506	0.400 ± 0.010	0.381–0.419	0.159 ± 0.005	0.149–0.169
lightgbm	0.772 ± 0.010	0.751–0.791	0.799 ± 0.009	0.780–0.815	0.426 ± 0.014	0.400–0.453	0.476 ± 0.016	0.446–0.508	0.419 ± 0.012	0.396–0.443	0.156 ± 0.005	0.146–0.166
p value	p < 0.001		p < 0.01		p < 0.05		p < 0.001		p < 0.001		p < 0.01	
Machine learning algorithms												
AdaBoost	0.832 ± 0.007	0.817–0.846	0.838 ± 0.003	0.832–0.844	0.458 ± 0.010	0.438–0.477	0.525 ± 0.017	0.491–0.554	0.487 ± 0.012	0.464–0.509	0.215 ± 0.002	0.211–0.219
BernoulliNB	0.743 ± 0.019	0.704–0.777	0.752 ± 0.020	0.707–0.785	0.338 ± 0.015	0.308–0.365	0.553 ± 0.035	0.485–0.618	0.399 ± 0.018	0.363–0.432	0.172 ± 0.007	0.159–0.186
CatBoost	0.872 ± 0.005	0.862–0.881	0.872 ± 0.002	0.867–0.877	0.600 ± 0.012	0.576–0.624	0.430 ± 0.018	0.395–0.468	0.491 ± 0.014	0.464–0.517	0.094 ± 0.001	0.091–0.096
DecisionTree	0.628 ± 0.009	0.611–0.645	0.800 ± 0.004	0.792–0.809	0.342 ± 0.012	0.317–0.365	0.383 ± 0.018	0.348–0.415	0.358 ± 0.013	0.332–0.384	0.200 ± 0.004	0.191–0.208
ExtraTree	0.852 ± 0.006	0.840–0.862	0.858 ± 0.004	0.851–0.864	0.571 ± 0.033	0.508–0.635	0.267 ± 0.023	0.222–0.314	0.332 ± 0.025	0.282–0.382	0.099 ± 0.002	0.096–0.102
GaussianNB	0.787 ± 0.008	0.770–0.803	0.798 ± 0.008	0.782–0.813	0.390 ± 0.012	0.366–0.413	0.580 ± 0.026	0.526–0.630	0.455 ± 0.012	0.430–0.479	0.169 ± 0.008	0.154–0.186
GradientBoosting	0.866 ± 0.005	0.855–0.876	0.863 ± 0.004	0.856–0.870	0.536 ± 0.013	0.511–0.562	0.501 ± 0.019	0.463–0.541	0.515 ± 0.015	0.485–0.544	0.097 ± 0.002	0.094–0.100
KNeighbors	0.557 ± 0.012	0.532–0.579	0.623 ± 0.008	0.605–0.639	0.177 ± 0.009	0.159–0.194	0.419 ± 0.019	0.380–0.456	0.248 ± 0.012	0.225–0.271	0.263 ± 0.005	0.252–0.273
LogisticRegression	0.782 ± 0.009	0.764–0.799	0.750 ± 0.005	0.740–0.760	0.334 ± 0.008	0.318–0.349	0.699 ± 0.019	0.662–0.735	0.451 ± 0.011	0.430–0.471	0.180 ± 0.004	0.173–0.187
MLP	0.743 ± 0.009	0.724–0.761	0.777 ± 0.016	0.744–0.806	0.390 ± 0.021	0.349–0.433	0.516 ± 0.025	0.468–0.564	0.414 ± 0.014	0.387–0.441	0.178 ± 0.016	0.151–0.212
NGBoost	0.881 ± 0.004	0.873–0.889	0.835 ± 0.005	0.825–0.844	0.463 ± 0.010	0.444–0.483	0.680 ± 0.016	0.648–0.711	0.548 ± 0.010	0.527–0.568	0.114 ± 0.002	0.110–0.117
RandomForest	0.870 ± 0.005	0.860–0.880	0.862 ± 0.003	0.856–0.868	0.564 ± 0.016	0.532–0.596	0.363 ± 0.020	0.326–0.402	0.427 ± 0.017	0.396–0.459	0.097 ± 0.001	0.095–0.100
SVM	0.549 ± 0.011	0.527–0.572	0.585 ± 0.023	0.540–0.630	0.184 ± 0.013	0.160–0.210	0.430 ± 0.024	0.382–0.481	0.240 ± 0.009	0.222–0.258	0.240 ± 0.002	0.235–0.244
XGBoost	0.846 ± 0.008	0.830–0.860	0.858 ± 0.003	0.852–0.863	0.524 ± 0.012	0.501–0.547	0.407 ± 0.021	0.365–0.450	0.449 ± 0.016	0.417–0.479	0.112 ± 0.002	0.107–0.116
p value	p < 0.001		p < 0.001		p < 0.001		p < 0.001		p < 0.001		p < 0.001	

**TABLE 4:** Sensitivity analysis of model performance after variable exclusion.

Variable	Excluded	AUC	Accuracy	Precision	Recall	F1	Brier
Base_ALL		0.896 ± 0.013	0.855 ± 0.023	0.510 ± 0.054	0.755 ± 0.060	0.607 ± 0.045	0.11 ± 0.013
Recurrent nephrotic syndrome	Yes	0.885 ± 0.013	0.827 ± 0.018	0.447 ± 0.037	0.702 ± 0.067	0.545 ± 0.040	0.125 ± 0.012
		p < 0.01	p < 0.01	p < 0.01	p < 0.01	p < 0.01	p < 0.01
DD	Yes	0.856 ± 0.016	0.868 ± 0.020	0.555 ± 0.068	0.589 ± 0.066	0.568 ± 0.047	0.102 ± 0.008
		p < 0.01	p < 0.01	p < 0.01	p < 0.01	p < 0.01	p < 0.01
umALB/Ucr	Yes	0.895 ± 0.012	0.857 ± 0.022	0.514 ± 0.052	0.752 ± 0.063	0.609 ± 0.045	0.109 ± 0.012
		p > 0.05	p < 0.01	p < 0.05	p > 0.05	p > 0.05	p < 0.01
Statins	Yes	0.903 ± 0.013	0.855 ± 0.025	0.512 ± 0.059	0.754 ± 0.063	0.607 ± 0.046	0.107 ± 0.013
		p < 0.01	p > 0.05	p > 0.05	p > 0.05	p > 0.05	p < 0.01
ALB	Yes	0.899 ± 0.013	0.863 ± 0.022	0.529 ± 0.055	0.764 ± 0.058	0.623 ± 0.044	0.105 ± 0.012
		p < 0.01	p < 0.01	p < 0.01	p < 0.05	p < 0.01	p < 0.01
CHE	Yes	0.893 ± 0.014	0.859 ± 0.021	0.518 ± 0.050	0.730 ± 0.061	0.604 ± 0.042	0.109 ± 0.011
		p < 0.01	p < 0.01	p < 0.01	p < 0.01	p > 0.05	p < 0.05
FDP > 5 mg/L	Yes	0.883 ± 0.013	0.839 ± 0.020	0.475 ± 0.040	0.765 ± 0.056	0.585 ± 0.038	0.124 ± 0.011
		p < 0.01	p < 0.01	p < 0.01	p < 0.01	p < 0.01	p < 0.01
INR	Yes	0.887 ± 0.015	0.829 ± 0.024	0.452 ± 0.049	0.700 ± 0.071	0.548 ± 0.048	0.118 ± 0.014
		p < 0.01	p < 0.01	p < 0.01	p < 0.01	p < 0.01	p < 0.01
aPLA2Rab	Yes	0.894 ± 0.013	0.851 ± 0.023	0.500 ± 0.051	0.744 ± 0.059	0.596 ± 0.044	0.113 ± 0.013
		p < 0.05	p < 0.01	p < 0.01	p < 0.01	p < 0.01	p < 0.01
AT III activity	Yes	0.897 ± 0.012	0.856 ± 0.020	0.513 ± 0.050	0.726 ± 0.062	0.599 ± 0.042	0.109 ± 0.011
		p < 0.05	p > 0.05	p > 0.05	p < 0.01	p < 0.01	p < 0.05

**TABLE 5:** The evaluation results of models based on test set.

Models	Algorithms	Fill	Sampling	Selection	Accuracy	AUC	Precision	Recall	Brier	F1
Optimal Model	NGBoost	KNN	BSMOTE	Freq-based	0.876	0.911	0.556	0.789	0.095	0.652
model_2	RF	KNN	SMOTE	LightGBM	0.868	0.918	0.750	0.158	0.084	0.261
model_3	NGBoost	RF	BSMOTE	ALL	0.853	0.913	0.500	0.632	0.097	0.558
model_4	LR	KNN	BSMOTE	freq-based	0.767	0.836	0.366	0.789	0.153	0.500
model_5	RF	RF	BSMOTE	LightGBM	0.915	0.888	0.750	0.632	0.088	0.686

NGBoost, Natural Gradient Boosting; LR, logistic regression; KNN, K-Nearest Neighbors; RF, random forest; SMOTE, synthetic minority oversampling Technique; BSMOTE, borderline-SMOTE; LightGBM, light gradient boosting machine; Freq-based, Frequency-based.

Decision curve analysis (DCA) assesses the clinical value of a prediction model by estimating its net benefit across various decision thresholds. The threshold probability is the minimum probability of a positive outcome at which a clinician would initiate an intervention (e.g., a threshold of 0.3 implies initiating prophylactic anticoagulation if the predicted probability is ≥0.3). In our analysis, the decision curve exceeded the “treat all” and “treat none” benchmarks for threshold probabilities between 0.2 and 0.9. This demonstrates that following the model’s recommendations provides a superior net benefit, quantifying the clinical gains after weighing the risks and benefits, compared to blanket clinical strategies over this probability range.

The optimal model was compared with the Padua score, a common VTE risk assessment tool for medical inpatients, using the test set from this study. Patients with a Padua score ≥4 were classified as high-risk. In the ROC comparison, the optimal model’s curve remained above that of the Padua score, with AUCs of 0.911 and 0.564, respectively. The confusion matrices further highlight that the performance gap primarily stems from the identification of high-risk patients. The optimal model identified 15 true positives, compared to only six by the Padua score.

Stratified sampling with replacement was performed multiple times to draw subsamples representing varying proportions (10%–100%) of the total sample size. The AUC was calculated for each subsample. The mean AUC and standard deviation were then derived across all iterations at each sampling proportion. Error bars in Figure 6 represent the standard deviation of the AUC estimates at their respective sampling proportion.

### 3.5 Web-based prediction tool

**FIGURE 7:**
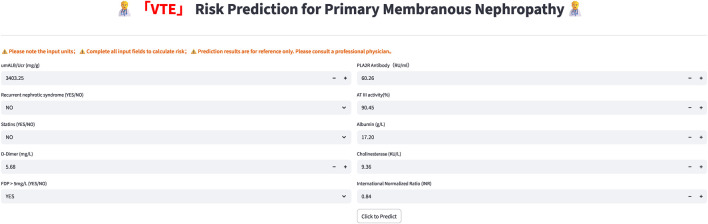
Illustration of the Webpage prediction interface.

**FIGURE 8:**
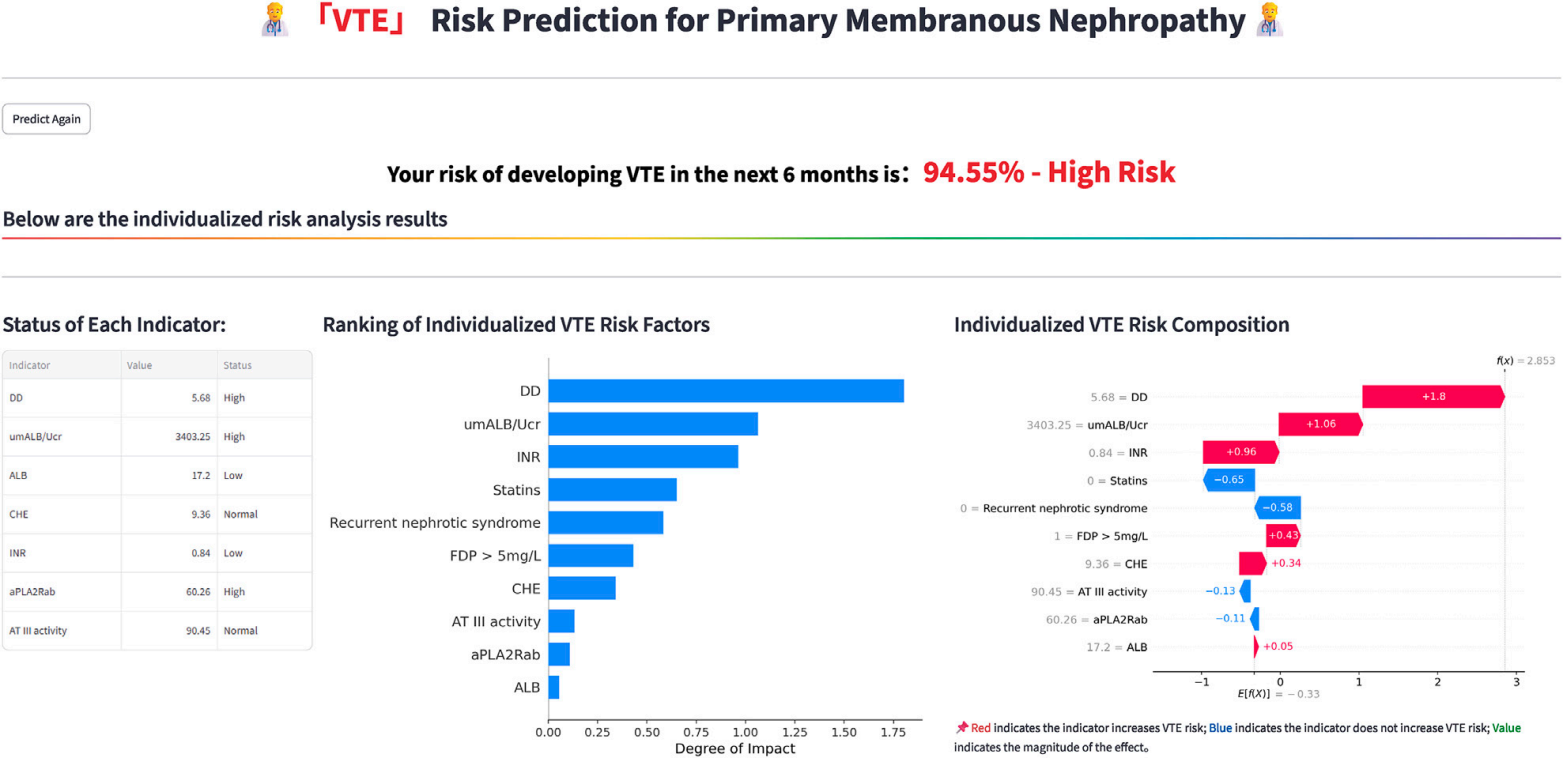
A display of the prediction result.

Based on the aforementioned results, the functionality of rapidly constructing web pages using Streamlit was utilized to develop the optimal model of this study into a web-based prediction tool. The input and output interfaces of this tool are depicted in Figures 7, 8. The model has been deployed on a cloud server and is publicly accessible via the following link: https://predict-pmn-s-vte-risk-arvbrnedu7g8ehqolwpje2.streamlit.app/.

## 4 Discussion

PMN accounts for approximately 80% of cases of MN and is a common renal disease characterized by prominent symptoms of proteinuria and hypoalbuminemia (Couser, 2017). The incidence of PMN in China has been increasing annually (Hou et al., 2018). VTE represents one of the most frequent complications of PMN, encompassing RVT, DVT, and PE. VTE often presents insidiously and poses serious risks, potentially exacerbating the condition of PMN patients, necessitating appropriate VTE prevention strategies. Key to VTE prevention is accurate risk prediction and the implementation of rational preventive measures. Currently, no specific VTE risk prediction model exists for PMN patients. Therefore, this study aims to construct a VTE risk prediction model using machine learning algorithms for PMN patients, providing essential groundwork for prophylactic anticoagulation strategies.

This study investigated 86 characteristic variables among 643 PMN patients. A total of 36 datasets were generated using three data imputation methods, three data sampling methods, and three feature selection methods. Subsequently, 504 models were constructed using 14 machine learning algorithms. Univariate analysis revealed substantial impacts of data processing and modeling strategies on model performance. For data imputation, the KNN method achieved marginally higher AUC and F1-score than RF and simple mean imputation, suggesting its relative advantage in preserving data structure. Among sampling strategies, ADA and BSMOTE showed comparable discriminative ability, with only minor differences in recall and F1-score, indicating limited sensitivity to sampling techniques. Feature selection markedly influenced outcomes, where the frequent feature method outperformed alternatives (p < 0.001), supporting the value of retaining stable, high-frequency variables. Among machine learning algorithms, NGBoost, CatBoost, and RF delivered the strongest results, with NGBoost attaining the highest AUC (0.881 ± 0.004) and the lowest Brier score, highlighting its capacity for modeling complex relationships and providing well-calibrated probabilistic predictions. Overall, this study demonstrates that appropriate data preprocessing, feature selection, and algorithm choice critically influence the performance of VTE risk prediction models. In feature sensitivity analyses, we observed that exclusion of specific variables (such as statin use, ALB, and AT-III) slightly improved discriminative performance (e.g., AUC and F1-score), suggesting that these variables may introduce redundancy or noise, thereby increasing model variance and limiting generalizability. Nevertheless, given their established clinical relevance and plausibility, we retained these features in the final model to maintain interpretability and clinical completeness. These findings indicate that despite satisfactory overall performance, some feature redundancy or multicollinearity may persist. Future studies should further optimize and validate the model using larger, multi-center datasets.

For the risk prediction of VTE formation, current clinical practice employs VTE scoring systems. Examples include the (Caprini et al., 1991; Barbar et al., 2010), and (Khorana et al., 2008) scores. However, the Caprini and Khorana scores are generally not applicable to PMN patients, and the Padua score does not incorporate PMN-specific predictors for VTE risk, such as hypoalbuminemia, proteinuria, and aPLA2Rab. The Padua score, refined by Barbar et al., in 2010 and validated in medical inpatients, remains one of the most widely used VTE risk assessment tools in clinical practice. Given that PMN patients align closely with the target population for this score, we compared our model against it. Results demonstrated superior performance of our optimal model in the test set. This finding is consistent with a study of 3,277 patients hospitalized with acute exacerbation of COPD, where the Padua score also underperformed relative to the Caprini score (AUC:0.644 ± 0.023 vs.0.713 ± 0.021, p = 0.029) (Zhou et al., 2022). Yang et al. reported similar findings. Padua score demonstrates limited predictive performance in PMN patients, which primarily stems from the score’s lack of specificity for this population. The 11 Padua items do not capture several PMN-specific risk factors, such as the disease itself being an independent VTE risk factor, along with hypoalbuminemia, heavy proteinuria, and hyperlipidemia. Furthermore, certain items such as “reduced mobility” often do not align with the clinical status of PMN patients. The high weight assigned to this item further reduces the score’s applicability. Additionally, some PMN patients with severe hypoalbuminemia receive prophylactic anticoagulation, which may alter their actual VTE risk. In summary, the limited clinical relevance of several key Padua items in the PMN population contributes to its suboptimal predictive performance. Another significant study was conducted by Lee et al., in 2014 (Lee et al., 2014), who developed a Markov model integrating serum albumin levels, bleeding risks, and acceptable risk-benefit ratios to quantify the potential benefit of prophylactic anticoagulation with warfarin in MN patients. It is important to note that this model is applicable only to MN patients receiving warfarin and incorporates serum albumin levels as the sole risk factor for VTE. Additionally, some scholars argue that the HAS-BLED score may be more suitable for assessing bleeding risk (Li et al., 2023). These factors significantly limit its generalizability and external applicability. Furthermore, clinical decision algorithms published to guide prophylactic anticoagulation in MN similarly rely solely on serum albumin levels to assess VTE risk (Hofstra and Wetzels, 2016; Lin et al., 2020; Rovin et al., 2021). This study focusses on VTE risk in MN patients, addressing key issues in prophylactic anticoagulation by integrating multiple factors and overcoming limitations associated with anticoagulant use.

The optimal model ultimately incorporated ten clinical indicators associated with VTE in PMN, including DD, FDP >5 mg/L, AT-Ⅲ, ALB, and six additional validated predictors. This suggests that in making decisions regarding prophylactic anticoagulation therapy for PMN patients, not only ALB levels but also the aforementioned indicators may be valuable considerations. The elevation of DD levels is closely associated with a hypercoagulable state and increased fibrinolysis in the body (Tripodi, 2011). SHAP results indicate that lower levels of DD are associated with lower risks of VTE, which aligns with findings from Li’s study (Li et al., 2012). As a terminal product of fibrinolysis, FDP becomes elevated during thrombosis and dissolution, similar to DD. Catalina Filip et al. reported higher FDP levels in pregnant women with DVT compared to non-DVT controls in the third trimester (Filip et al., 2025). Similarly, Zheng et al. identified FDP as an independent risk factor and incorporated it into a nomogram for preoperative DVT risk in lower limb fracture patients (Zheng et al., 2024). Consistent with these findings, SHAP analysis in our model indicated that FDP levels >5 mg/L contributed to increased predicted VTE risk. The SHAP analysis similarly underscore the significant role of umALB/Ucr in the model’s predictions: higher levels are associated with an increased predicted risk of VTE. This finding is consistent with the large-scale cohort study by Massicotte-Azarniouch et al., which reported that even in individuals with normal renal function, heavy albuminuria significantly elevates the risk of VTE and was identified as an independent risk factor (Massicotte-Azarniouch et al., 2017). On the other hand, SHAP analysis suggested statin use was associated with higher predicted VTE risk. This likely reflects confounding by indication, patients already prescribed statins before admission may have had higher baseline risk. While medication history is essential for modeling, such reverse causality should be considered in interpretation. AT-III is the principal synthesized component of the anticoagulant system in the liver, providing 70% of the body’s anticoagulant activity. SHAP analysis indicates that decreased AT-III activity is associated with an increased risk of VTE. Huang et al. found that patients with PMN in a hypercoagulable state have lower AT-III activity compared to those without hypercoagulability (Huang et al., 2016). Serum albumin levels are recognized as a risk factor for VTE in PMN patients. Consistently, the SHAP analysis in this study suggests that low serum albumin levels are similarly associated with a heightened risk of VTE.

## 5 Limitations

This study is a single-center retrospective study, and the retrospective design inherently introduces certain biases, particularly in the accuracy of data recording and patient selection. First, the retrospective nature of data collection lacks real-time precision and may result in incomplete or inaccurate information, especially in the absence of standardized recording procedures. Although various measures were implemented during the data preprocessing to mitigate these biases, such as feature selection and techniques for handling missing data, we acknowledge that these efforts cannot entirely eliminate all limitations. In the collection of key variables, we encountered the absence of critical risk factor data, such as the Leiden factor, total plasma protein S, and prothrombin gene mutations. Such deficiency could potentially compromise the accuracy of the model’s predictions. Additionally, data selection was constrained by the retrospective nature of the study. The majority of patients had multiple clinical visits on record. Given that this study aims to predict risk and implement early VTE prevention interventions while accounting for confounding factors, we chose to include only the data from patients’ initial visits. The rationale is that first-visit data more accurately reflect patients’ real health status before substantial medical intervention. This decision, while helpful in reducing confounding factors arising from variations and interventions during treatment, also introduces a series of limitations, particularly in terms of data integrity and the comprehensiveness of disease progression information. For example, it fails to reflect the evolution of the patient’s condition, lacks in-depth assessment of the patient’s health status, and does not incorporate adjustments in treatment strategies into the model developing, thereby affecting the reliability and accuracy of the model’s predictions. Finally, the sample size in this study was relatively limited, with only 643 PMN patients included, among whom just 93 were VTE-positive. Small sample size and class imbalance pose challenges to the model’s ability to handle rare events and conduct subgroup analyses. Although techniques such as SMOTE and Borderline-SMOTE were applied to address class imbalance, the limited sample size may still constrain the model’s generalizability in clinical practice. Moreover, as this study employed a single-center retrospective design, the data were restricted to cases and patient characteristics from a specific medical center, potentially limiting the external validity and generalizability of the findings. The model’s generalization capability has not been extensively validated due to the singularity of the data source, and its applicability in different populations and healthcare settings requires further investigation. Data from a single center may not fully represent all patient groups, particularly those from diverse regions, ethnicities, or healthcare systems. The model specifically predicts symptomatic VTE risk, as only clinically symptomatic cases underwent confirmatory imaging. Asymptomatic VTE cases may have been undetected due to the absence of systematic screening.

To address the current limitations of the study and further validate the model’s performance, we propose several directions for future work. First, designing and conducting prospective studies. Compared to retrospective studies, prospective studies provide more accurate data by allowing standardized and on-demand data collection from the outset, thereby reducing the risks of missing data and information bias, which enhances the reliability of research findings and the precision of model predictions. Second, delving into the methods for processing and integrating multiple hospitalization records by developing time-series models or dynamic prediction models to meticulously consider detailed information from each patient encounter, constructing a continuous risk prediction trajectory for patients, and monitoring the temporal variations in patients’ VTE risks. Third, collaborating with other healthcare institutions to establish a multi-center database. By incorporating data from diverse regions and populations, this strategy significantly increases sample size, improves the study’s statistical ability, and enables a more comprehensive evaluation of the model’s generalizability. It also ensures its applicability and effectiveness across various healthcare settings. Through these efforts, we aim to overcome the limitations of single-center, retrospective studies and provide robust evidence for the application of the risk prediction model in real-world clinical practice, ultimately contributing to better patient outcomes.

## 6 Conclusion

This study utilized a single-center dataset, employing advanced statistical methods and machine learning techniques to develop and evaluate a personalized VTE risk prediction model for PMN patients. A web-based prediction tool was also created. As a single-center retrospective study, certain limitations exist, including a relatively small sample size, imbalanced sample categories, and missing data. However, data preprocessing measures such as feature selection and sample resampling were employed to significantly reduce the biases introduced by these limitations. Internal validation of the model demonstrated good predictive performance, suggesting its potential to assist in VTE prevention and management for PMN patients. Looking forward, we aim to address these limitations by conducting prospective trials or collaborating with other institutions to construct multi-center datasets, thereby paving the way for further development and broader application of the model.

## Glossary

AdaBoost: Adaptive Boosting
ADASYN: Adaptive Synthetic
AFP: alpha fetoprotein
A/G: albumin/globulin
ALB: albumin
ALT: alanine transaminase
ALP: alkaline phosphatase
APTR: activated partial thromboplastin ratio
aPLA2Rab: anti-phospholipase A2 receptor antibody
AST: aspartate aminotransferase
AT III: antithrombin III
AUC: area under the curve
Ber NB: Bernoulli naive Bayes
BNP: brain natriuretic peptide
CA: carbohydrate antigens
CatBoost: Categorical Boosting
CHE: cholinesterase
CK 19: cytokeratin 19
CK-MB: creatine kinase isoenzyme MB
Cl: chlorine
DCA: decision curve analysis
DD: D-dimer
DVT: deep venous thrombosis
eGFR: estimated glomerular filtration rate
EHRS: electronic health record system
EO%: eosinophil ratio
ESR: erythrocyte sedimentation rate
ExtRa Trees: Extremely Randomized Trees
FDP: fibrinogen degradation products
FOB: fecal occult blood
FIB: fibrinogen
Gau NB: Gaussian naive Bayes
GBoost: Gradient Boosting
GLB: globulin
Hcy: homocysteine
hs-CRP: hypersensitive-C reactive protein
INR: international normalized ratio
KNN: K-Nearest Neighbor
LDL-C: low-density lipoprotein cholesterol
LightGBM: Light Gradient Boosting Machine
Lp(a): lipoprotein a
LR: logistic regression
MCV: mean volume of red blood cells
MLP: multi-layer perceptron
MN: membranous nephropathy
MYO: myoglobin
NE: neutrophilic granulocyte
NEUT%: neutrophil ratio
NGBoost: Natural Gradient Boosting
PCT: procalcitonin
PE: pulmonary embolism
PLT: platelet count
PMN: primary membranous nephropathy
P-R: Precision-Recall
PT: prothrombin time
RF: random forest
ROC: receiver operating characteristic
RNS: relapse of nephrotic syndrome
RVT: renal vein thrombosis
SD: standard deviation
SHAP: shapley additive explanations
SMOTE: synthetic minority oversampling Technique
BSMOTE: borderline-SMOTE
SVM: support vector machine
TP: total protein
TT: thrombin time
UA: uric acid
uALB: urinary albumin
Ucr: urine creatinine
umALB: urinary microalbumin
VTE: venous thromboembolism
WBC: white blood cell
XGBoost: extreme Gradient Boosting.

## References

[B1] Barbar S. Noventa F. Rossetto V. Ferrari A. Brandolin B. Perlati M. (2010). A risk assessment model for the identification of hospitalized medical patients at risk for venous thromboembolism: the padua prediction score. J. Thrombosis Haemostasis JTH 8, 2450–2457. 10.1111/j.1538-7836.2010.04044.x 20738765

[B2] Bierzynska A. Saleem M. (2017). Recent advances in understanding and treating nephrotic syndrome. F1000Res 6, 121. 10.12688/f1000research.10165.1 28232870 PMC5302149

[B3] Caprini J. A. Arcelus J. I. Hasty J. H. Tamhane A. C. Fabrega F. (1991). Clinical assessment of venous thromboembolic risk in surgical patients. Semin. Thromb. Hemost. 17 (Suppl. 3), 304–312. 1754886

[B4] Cheung K. L. Zakai N. A. Callas P. W. Howard G. Mahmoodi B. K. Peralta C. A. (2018). Mechanisms and mitigating factors for venous thromboembolism in chronic kidney disease: the REGARDS study. J. Thromb. Haemost. 16, 1743–1752. 10.1111/jth.14235 29984467 PMC6123283

[B5] Couser W. G. (2017). Primary membranous nephropathy. Clin. J. Am. Soc. Nephrol. 12, 983–997. 10.2215/CJN.11761116 28550082 PMC5460716

[B6] Dc C. Pe B. (2017). Membranous nephropathy: integrating basic science into improved clinical management. Kidney Int. 91, 566–574. 10.1016/j.kint.2016.09.048 28065518

[B7] Filip C. Matasariu D. R. Ursache A. Dimitriu C. D. Filip C. Boiculese V. L. (2025). Exploring biomarkers to predict thrombogenic risk in pregnancy. J. Clin. Med. 14, 932. 10.3390/jcm14030932 39941603 PMC11818711

[B8] Hladunewich M. A. Troyanov S. Calafati J. Cattran D. C. (2009). The natural history of the non-nephrotic membranous nephropathy patient. Clin. J. Am. Soc. Nephrol. 4, 1417–1422. 10.2215/CJN.01330209 19661220 PMC2736692

[B9] Hofstra J. M. Wetzels J. F. M. (2016). Should aspirin be used for primary prevention of thrombotic events in patients with membranous nephropathy? Kidney Int. 89, 981–983. 10.1016/j.kint.2016.01.019 27083274

[B10] Hou J.-H. Zhu H.-X. Zhou M.-L. Le W.-B. Zeng C.-H. Liang S.-S. (2018). Changes in the spectrum of kidney diseases: an analysis of 40,759 biopsy-proven cases from 2003 to 2014 in China. Kidney Dis. (Basel) 4, 10–19. 10.1159/000484717 29594138 PMC5848489

[B11] Huang M. Wei R. Li Q. Yang X. Cao C. Su T. (2016). Hypercoagulable state evaluated by thromboelastography in patients with idiopathic membranous nephropathy. J. Thromb. Thrombolysis 41, 321–327. 10.1007/s11239-015-1247-x 26152497

[B12] Khorana A. A. Kuderer N. M. Culakova E. Lyman G. H. Francis C. W. (2008). Development and validation of a predictive model for chemotherapy-associated thrombosis. Blood 111, 4902–4907. 10.1182/blood-2007-10-116327 18216292 PMC2384124

[B13] Kumar S. Chapagain A. Nitsch D. Yaqoob M. M. (2012). Proteinuria and hypoalbuminemia are risk factors for thromboembolic events in patients with idiopathic membranous nephropathy: an observational study. BMC Nephrol. 13, 107. 10.1186/1471-2369-13-107 22963194 PMC3480900

[B14] Lee T. Biddle A. K. Lionaki S. Derebail V. K. Barbour S. J. Tannous S. (2014). Personalized prophylactic anticoagulation decision analysis in patients with membranous nephropathy. Kidney Int. 85, 1412–1420. 10.1038/ki.2013.476 24336031 PMC4040154

[B15] Li S.-J. Guo J.-Z. Zuo K. Zhang J. Wu Y. Zhou C. (2012). Thromboembolic complications in membranous nephropathy patients with nephrotic syndrome-a prospective study. Thromb. Res. 130, 501–505. 10.1016/j.thromres.2012.04.015 22633211

[B16] Li X. Xie X. Zhao Y. Wang G. Shao H. Zhang X. (2023). Some points for the KDIGO 2021 guideline for prophylactic anticoagulation in membranous nephropathy: is it clear enough for us to follow? Nephron 147, 193–198. 10.1159/000525913 35901785

[B17] Lin R. McDonald G. Jolly T. Batten A. Chacko B. (2020). A systematic review of prophylactic anticoagulation in nephrotic syndrome. Kidney Int. Rep. 5, 435–447. 10.1016/j.ekir.2019.12.001 32274450 PMC7136344

[B18] Lionaki S. Derebail V. K. Hogan S. L. Barbour S. Lee T. Hladunewich M. (2012). Venous thromboembolism in patients with membranous nephropathy. Clin. J. Am. Soc. Nephrol. 7, 43–51. 10.2215/CJN.04250511 22076873 PMC3265338

[B19] Mahmoodi B. K. ten Kate M. K. Waanders F. Veeger N. J. G. M. Brouwer J.-L. P. Vogt L. (2008). High absolute risks and predictors of venous and arterial thromboembolic events in patients with nephrotic syndrome: results from a large retrospective cohort study. Circulation 117, 224–230. 10.1161/CIRCULATIONAHA.107.716951 18158362

[B20] Massicotte-Azarniouch D. Bader Eddeen A. LazoLanger A. Molnar A. O. Lam N. N. McCallum M. K. (2017). Risk of venous thromboembolism in patients by Albuminuria and estimated GFR. Am. J. Kidney Dis. 70, 826–833. 10.1053/j.ajkd.2017.07.003 28823585

[B21] Ronco P. Debiec H. (2015). Pathophysiological advances in membranous nephropathy: time for a shift in patient’s care. Lancet 385, 1983–1992. 10.1016/S0140-6736(15)60731-0 26090644

[B22] Rovin B. H. Adler S. G. Barratt J. Bridoux F. Burdge K. A. Chan T. M. (2021). Executive summary of the KDIGO 2021 Guideline for the Management of Glomerular Diseases. Kidney Int. 100, 753–779. 10.1016/j.kint.2021.05.015 34556300

[B23] Singhal R. Brimble K. S. (2006). Thromboembolic complications in the nephrotic syndrome: pathophysiology and clinical management. Thromb. Res. 118, 397–407. 10.1016/j.thromres.2005.03.030 15990160

[B24] Tripodi A. (2011). D-dimer testing in laboratory practice. Clin. Chem. 57, 1256–1262. 10.1373/clinchem.2011.166249 21719689

[B25] Velasquez Forero F. Garcia Prugue N. Ruiz Morales N. (1988). Idiopathic nephrotic syndrome of the adult with asymptomatic thrombosis of the renal vein. Am. J. Nephrol. 8, 457–462. 10.1159/000167654 3218659

[B26] Vestergaard S. V. Birn H. Darvalics B. Nitsch D. Sørensen H. T. Christiansen C. F. (2022). Risk of arterial thromboembolism, venous thromboembolism, and bleeding in patients with nephrotic syndrome: a population-based cohort Study. Am. J. Med. 135, 615–625.e9. 10.1016/j.amjmed.2021.11.018 34979093

[B27] Wu H. H. L. Alozai A. Li J. W. C. Elmowafy A. Ponnusamy A. Woywodt A. (2022). Risk factors of venous thromboembolism in anti-PLA2R-positive and negative primary membranous nephropathy. Clin. Kidney J. 15, 1636–1638. 10.1093/ckj/sfac052 35892019 PMC9308090

[B28] Zheng F. Chen X. Huang J. Lin C. (2024). Study on risk factors of preoperative deep vein thrombosis in patients with lower limb fractures and construction and validation of risk prediction nomogram model. BMC Surg. 24, 408. 10.1186/s12893-024-02718-3 39709353 PMC11662550

[B29] Zhou C. Yi Q. Ge H. Wei H. Liu H. Zhang J. (2022). Validation of risk assessment models predicting venous thromboembolism in inpatients with acute exacerbation of chronic obstructive pulmonary disease: a multicenter cohort Study in China. Thromb. Haemost. 122, 1177–1185. 10.1055/a-1693-0063 34758489

[B30] Zhu H. Xu L. Liu X. Liu B. Zhai C. Wang R. (2022). Anti-PLA2R antibody measured by ELISA predicts the risk of vein thrombosis in patients with primary membranous nephropathy. Ren. Fail 44, 594–600. 10.1080/0886022X.2022.2057861 35380081 PMC8986254

