# Supplementary material for: Development of a venous thromboembolism risk prediction model for patients with primary membranous nephropathy based on machine learning
Source: Front Pharmacol. 2025 Nov 6;16:1683708. doi: 10.3389/fphar.2025.1683708 (PMC12631078; doi:10.3389/fphar.2025.1683708)
Supplement: Supplementary file 1 [file Supplementaryfile1.docx]

**Supplementary Tables**

S Tab 1 Basic information Characteristics Between the VTE Group and Non-VTE Group

S Tab 2 Medical History and Complications Characteristics Between the VTE Group and Non-VTE Group

S Tab 3 Medication Characteristics Between the VTE Group and Non-VTE Group

S Tab 4 Laboratory Test indicator Characteristics Between the VTE Group and Non-VTE Group

S Tab 5 Multivariable Analysis of VTE Risk Factors

S Tab 6 Included variables

S Tab 7 missing characteristics of each variable

S Tab 8 Medical Reference Ranges for Discretized Features and Model-Incorporated Characteristics

S Tab 9 Summary of Composite Feature

S Tab 1 Basic information Characteristics Between the VTE Group and Non-VTE Group

| **Variables** | **Non VTE patients** | **VTE patients** | **P value** |
| --- | --- | --- | --- |
| Age | 53.30 (14.24) | 58.77 (14.64) | 0.001 |
| Height | 162.73 (7.18) | 162.75 (7.25) | 0.980 |
| Weight | 66.78 (13.19) | 66.47 (12.22) | 0.818 |
| BMI | 25.17 (4.58) | 25.00 (3.84) | 0.704 |
| Gender |  |  |  |
| Female | 196(35.64%) | 26(30.95%) | 0.474 |
| Male | 354(64.36%) | 58(69.05%) |  |
| Ethnic |  |  |  |
| Han | 503 (91.45%) | 84 (90.32%) | 0.570 |
| Tibetan | 29 (5.27%) | 4 (4.30%) |  |
| Yi | 6 (1.09%) | 3 (3.23%) |  |
| Hui | 1 (0.18%) |  |  |
| other | 11 (2.00%) | 2 (2.15%) |  |
| Smoking history exceeding 10 years |  |  |  |
| no | 525 (95.45%) | 86 (92.47%) | 0.205 |
| yes | 25 (4.55%) | 7 (7.53%) |  |
| Alcohol consumption history exceeding 10 years |  |  |  |
| no | 538 (97.82%) | 85 (91.40%) | 0.004 |
| yes | 12 (2.18%) | 8 (8.60%) |  |
| Less than 6 months of PMN duration |  |  |  |
| no | 190 (34.55%) | 29 (31.18%) | 0.607 |
| yes | 360 (65.45%) | 64 (68.82%) |  |

S Tab 2 Medical History and Complications Characteristics Between the VTE Group and Non-VTE Group

| **Variables** | **Non VTE patients** | **VTE patients** | **P value** |
| --- | --- | --- | --- |
| Respiratory failure |  |  |  |
| no | 541 (98.36%) | 90 (96.77%) | 0.395 |
| yes | 9 (1.64%) | 3 (3.23%) |  |
| heart failure |  |  |  |
| no | 541 (98.36%) | 91 (97.85%) | 0.665 |
| yes | 9 (1.64%) | 2 (2.15%) |  |
| Cerebral infarction or myocardial infarction |  |  |  |
| no | 511 (92.91%) | 85 (91.40%) | 0.762 |
| yes | 39 (7.09%) | 8 (8.60%) |  |
| Hypercoagulability |  |  |  |
| no | 535 (97.27%) | 90 (96.77%) | 0.735 |
| yes | 15 (2.73%) | 3 (3.23%) |  |
| Lung diseases |  |  |  |
| no | 417 (75.82%) | 67 (72.04%) | 0.515 |
| yes | 133 (24.18%) | 26 (27.96%) |  |
| arteriosclerosis |  |  |  |
| no | 467 (84.91%) | 64 (68.82%) | <0.001 |
| yes | 83 (15.09%) | 29 (31.18%) |  |
| Metabolic Dysregulation Syndrome |  |  |  |
| no | 534 (97.09%) | 90 (96.77%) | 0.747 |
| yes | 16 (2.91%) | 3 (3.23%) |  |
| Hypertension |  |  |  |
| no | 285 (51.82%) | 43 (46.24%) | 0.377 |
| yes | 265 (48.18%) | 50 (53.76%) |  |
| Diabetes |  |  |  |
| no | 466 (84.73%) | 70 (75.27%) | 0.034 |
| yes | 84 (15.27%) | 23 (24.73%) |  |
| Electrolyte imbalances |  |  |  |
| no | 525 (95.45%) | 80 (86.02%) | 0.001 |
| yes | 25 (4.55%) | 13 (13.98%) |  |
| Acute illness |  |  |  |
| no | 483 (87.82%) | 70 (75.27%) | 0.002 |
| yes | 67 (12.18%) | 23 (24.73%) |  |
| Hyperlipidemia |  |  |  |
| no | 348 (63.27%) | 58 (62.37%) | 0.959 |
| yes | 202 (36.73%) | 35 (37.63%) |  |
| Liver diseases |  |  |  |
| no | 426 (77.45%) | 66 (70.97%) | 0.218 |
| yes | 124 (22.55%) | 27 (29.03%) |  |
| Hyperuricemia |  |  |  |
| no | 449 (81.64%) | 71 (76.34%) | 0.290 |
| yes | 101 (18.36%) | 22 (23.66%) |  |
| Infectious diseases |  |  |  |
| no | 449 (81.64%) | 61 (65.59%) | 0.001 |
| yes | 101 (18.36%) | 32 (34.41%) |  |
| Surgery within the past month |  |  |  |
| no | 264 (48.00%) | 42 (45.16%) | 0.693 |
| yes | 286 (52.00%) | 51 (54.84%) |  |
| Active cancer |  |  |  |
| no | 542 (98.55%) | 91 (97.85%) | 0.644 |
| yes | 8 (1.45%) | 2 (2.15%) |  |
| Lung infections |  |  |  |
| no | 465 (84.55%) | 63 (67.74%) | <0.001 |
| yes | 85 (15.45%) | 30 (32.26%) |  |
| Recurrent nephrotic syndrome |  |  |  |
| no | 373 (69.46%) | 42 (45.16%) | <0.001 |
| yes | 164 (30.54%) | 51 (54.84%) |  |
| History of cancer |  |  |  |
| no | 526 (95.64%) | 90 (96.77%) | 0.784 |
| yes | 24 (4.36%) | 3 (3.23%) |  |
| History of VTE |  |  |  |
| no | 532 (96.73%) | 86 (92.47%) | 0.074 |
| yes | 18 (3.27%) | 7 (7.53%) |  |

S Tab 3 Medication Characteristics Between the VTE Group and Non-VTE Group

| **Variables** | **Non VTE patients** | **VTE patients** | **P value** |
| --- | --- | --- | --- |
| Antiplatelet drugs |  |  |  |
| no | 509 (92.55%) | 84 (90.32%) | 0.595 |
| yes | 41 (7.45%) | 9 (9.68%) |  |
| Diuretics |  |  |  |
| no | 364 (66.18%) | 33 (35.48%) | <0.001 |
| yes | 186 (33.82%) | 60 (64.52%) |  |
| Statins |  |  |  |
| no | 250 (45.45%) | 29 (31.18%) | 0.014 |
| yes | 300 (54.55%) | 64 (68.82%) |  |
| Immunosuppressants |  |  |  |
| no | 151 (27.45%) | 17 (18.28%) | 0.083 |
| yes | 399 (72.55%) | 76 (81.72%) |  |
| Anticoagulants |  |  |  |
| no | 289 (52.55%) | 62 (66.67%) | 0.016 |
| yes | 261 (47.45%) | 31 (33.33%) |  |

S Tab 4 Laboratory Test indicator Characteristics Between the VTE Group and Non-VTE Group

| **Variables** | **Non VTE patients** | **VTE patients** | **P value** |
| --- | --- | --- | --- |
| Hematocrit | 38.62 (8.63) | 37.08 (7.66) | 0.082 |
| Haemoglobin | 130.82 (22.44) | 123.90 (22.45) | 0.007 |
| MCV | 91.21 (6.22) | 91.03 (7.39) | 0.826 |
| PLT | 228.28 (81.72) | 234.81 (127.78) | 0.634 |
| NE | 5.29 (2.70) | 5.12 (2.09) | 0.482 |
| NEUT% | 68.00 [60.08,74.90] | 70.60 [62.30,76.10] | 0.316 |
| WBC | 7.53 (2.79) | 7.37 (2.51) | 0.594 |
| EO% | 1.50 [0.70,2.70] | 1.40 [0.40,3.10] | 0.686 |
| A/G | 1.15 (0.31) | 0.91 (0.32) | <0.001 |
| AST/ALT | 1.50 [1.10,1.90] | 1.80 [1.40,2.40] | <0.001 |
| ALB | 27.70 (7.40) | 21.54 (7.53) | <0.001 |
| ALB/uALB | 25.46 (65.69) | 10.71 (26.46) | <0.001 |
| GLB | 24.54 (4.85) | 24.08 (5.11) | 0.420 |
| AST | 28.00 [24.00,35.00] | 28.00 [23.00,38.00] | 0.482 |
| ALP | 76.58 (26.35) | 85.94 (40.93) | 0.035 |
| CHE | 10.85 (4.20) | 9.89 (3.43) | 0.018 |
| Cl | 107.34 (3.81) | 107.75 (4.04) | 0.364 |
| Urea | 5.80 [4.66,7.84] | 6.63 [5.35,9.83] | 0.002 |
| TP | 52.15 (10.27) | 45.62 (10.31) | <0.001 |
| UA | 378.69 (97.10) | 372.01 (102.29) | 0.559 |
| eGFR | 87.05 (30.06) | 76.52 (29.19) | 0.002 |
| CK-MB activity | 16.15 [12.22,21.60] | 17.50 [12.38,24.20] | 0.140 |
| Lp(a) | 329.00 [128.50,649.50] | 337.00 [173.50,620.50] | 0.421 |
| LDL-C | 3.95 [2.93,5.45] | 4.70 [3.23,5.94] | 0.012 |
| Hcy | 11.80 [9.30,15.05] | 11.80 [9.85,14.10] | 0.830 |
| APTR | 0.95 (0.10) | 0.96 (0.11) | 0.532 |
| TT | 17.80 [17.08,18.50] | 17.90 [16.90,18.40] | 0.707 |
| AT III activity | 91.37 (15.86) | 83.31 (16.39) | <0.001 |
| DD | 0.71 [0.39,1.50] | 2.69 [1.73,4.00] | <0.001 |
| FIB | 4.64 (1.53) | 5.34 (1.87) | 0.001 |
| PT | 10.91 (1.09) | 10.75 (2.05) | 0.451 |
| INR | 0.98 (0.11) | 0.96 (0.19) | 0.291 |
| CK 19 fragment | 2.20 [1.55,3.11] | 2.45 [1.65,3.29] | 0.165 |
| AFP | 3.46 (1.95) | 3.69 (3.35) | 0.524 |
| umALB | 3500.00 [1304.15,7900.00] | 4400.00 [2461.50,9800.00] | 0.013 |
| umALB/Ucr | 441.90 (434.23) | 927.31 (1496.35) | 0.003 |
| Ucr | 13116.14 (7604.74) | 13244.48 (7445.85) | 0.881 |
| 24h urine volume | 1.60 [1.20,2.00] | 1.47 [1.10,2.00] | 0.265 |
| 24h urinary protein | 4.40 [2.27,8.67] | 7.48 [4.28,10.65] | <0.001 |
| Urinary total protein | 3.03 [1.40,5.99] | 5.30 [2.74,8.90] | <0.001 |
| aPLA2Rab | 71.50 (159.51) | 141.19 (255.28) | 0.018 |
| ESR | 20.00 [2.90,44.00] | 35.00 [5.00,74.00] | 0.003 |
| BNP > 100pg/mL |  |  |  |
| no | 474 (86.18%) | 67 (72.04%) | 0.001 |
| yes | 76 (13.82%) | 26 (27.96%) |  |
| hs-CRP > 5mg/L |  |  |  |
| no | 461 (83.82%) | 66 (70.97%) | 0.005 |
| yes | 89 (16.18%) | 27 (29.03%) |  |
| MYO > 140ng/mL |  |  |  |
| no | 485 (88.18%) | 76 (81.72%) | 0.119 |
| yes | 65 (11.82%) | 17 (18.28%) |  |
| FDP > 5mg/L |  |  |  |
| no | 442 (80.36%) | 28 (31.11%) | <0.001 |
| yes | 108 (19.64%) | 62 (68.89%) |  |
| CA125 > 35U/mL |  |  |  |
| no | 354 (64.36%) | 27 (30.68%) | <0.001 |
| yes | 196 (35.64%) | 61 (69.32%) |  |
| PCT > 0.05ng/mL |  |  |  |
| no | 362 (65.82%) | 39 (42.39%) | <0.001 |
| yes | 188 (34.18%) | 53 (57.61%) |  |
| FOB |  |  |  |
| negative | 131 (23.95%) | 16 (17.20%) | 0.188 |
| weakly positive | 173 (31.63%) | 29 (31.18%) |  |
| positive | 156 (28.52%) | 36 (38.71%) |  |
| strongly positive | 87 (15.90%) | 12 (12.90%) |  |

AFP, alpha fetoprotein; A/G, albumin/globulin; ALB, albumin; ALT, alanine transaminase; ALP, alkaline phosphatase; aPLA2Rab, anti-phospholipase A2 receptor antibody; APTR, activated partial thromboplastin ratio; AST, aspartate aminotransferase; AT III, antithrombin III; BNP, brain natriuretic peptide; CA, carbohydrate antigens; CHE, cholinesterase; CK 19, cytokeratin 19; CK-MB, creatine kinase isoenzyme MB; Cl, chlorine; DD, D-dimer; GFR, estimated glomerular filtration rate; EO%, eosinophil ratio; ESR, erythrocyte sedimentation rate; FDP, fibrin degradation product; FOB, fecal occult blood; FIB, fibrinogen; GLB, globulin; Hcy, homocysteine; hs-CRP, hypersensitive-C reactive protein; INR, international normalized ratio; LDL-C, low-density lipoprotein cholesterol; Lp(a), lipoprotein a; MCV, mean volume of red blood cells; MYO, myoglobin; NE, neutrophilic granulocyte; NEUT%, neutrophil ratio; PCT, procalcitonin; PLT, platelet count; PT, prothrombin time; SCr, creatinine; TP, total protein; TT, thromboplastin time; UA, uric acid; uALB, urinary albumin; Ucr, urine creatinine; umALB, urinary microalbumin; WBC, white blood cell;

S Tab 5 Multivariable Analysis of VTE Risk Factors

| **Variables** | **coef** | **std err** | **z** | **p** | **95%Cl** | |
| --- | --- | --- | --- | --- | --- | --- |
| **Recurrent nephrotic syndrome** | **0.8063** | **0.296** | **2.721** | **0.007** | **0.225** | **1.387** |
| **Anticoagulants** | **0.8734** | **0.338** | **2.581** | **0.010** | **0.210** | **1.536** |
| **AT III activity** | **-0.0196** | **0.009** | **-2.067** | **0.039** | **-0.038** | **-0.001** |
| **DD** | **0.0750** | **0.036** | **2.091** | **0.037** | **0.005** | **0.145** |
| **FDP > 5mg/L** | **1.2086** | **0.322** | **3.752** | **0.000** | **0.577** | **1.840** |
| **PCT > 0.05ng/mL** | **0.7503** | **0.279** | **2.686** | **0.007** | **0.203** | **1.298** |

S Tab 6 Included variables

| **Categories** | **Variables** |
| --- | --- |
| Basic information (9) | age, height, weight, BMI, gender, ethnic, smoking history exceeding 10 years, alcohol consumption history exceeding 10 years, PMN duration |
| Medical History and Complications (21) | Respiratory failure, heart failure, Cerebral infarction or myocardial infarction, Hypercoagulability, Lung diseases, arteriosclerosis, Metabolic Dysregulation Syndrome, Hypertension, Diabetes, Electrolyte imbalances, Acute illness, Hyperlipidemia, Liver diseases, Hyperuricemia, Infectious diseases, Surgery within the past month, Active cancer, Lung infections, Recurrent nephrotic syndrome, History of cancer, History of VTE |
| Medication (5) | antiplatelet drugs, diuretics, statins, immunosuppressants, anticoagulants |
| Laboratory Test indicator (51) | Variables, Hematocrit, Haemoglobin, MCV, PLT, NE, NEUT%, WBC, EO%, A/G, AST/ALT, ALB, ALB/uALB, GLB, AST, ALP, CHE, Cl, SCr, Urea, TP, Urea/SCr, UA, eGFR, CK-MB activity, Lp(a), LDL-C, Hcy, APTR, TT, AT III activity, DD, FIB, PT, INR, CK 19 fragment, AFP, umALB, umALB/Ucr, Ucr, 24h urine volume, 24h urinary protein, urinary total protein, aPLA2Rab, ESR, BNP > 100pg/mL, hs-CRP > 5mg/L, MYO > 140ng/mL, FDP > 5mg/L, CA125 > 35U/mL, PCT > 0.05ng/mL, FOB |

S Tab 7 missing characteristics of each variable

| **Variables** | **Non-missing numbers** | **Missing numbers** | **Missing ratio** |
| --- | --- | --- | --- |
| gender | 643 | 0 | 0.00% |
| age | 643 | 0 | 0.00% |
| height | 643 | 0 | 0.00% |
| weight | 643 | 0 | 0.00% |
| BMI | 643 | 0 | 0.00% |
| ethnic | 643 | 0 | 0.00% |
| Respiratory failure | 643 | 0 | 0.00% |
| Heart failure | 643 | 0 | 0.00% |
| Cerebral infarction or myocardial infarction | 643 | 0 | 0.00% |
| Hypercoagulability | 643 | 0 | 0.00% |
| Lung diseases | 643 | 0 | 0.00% |
| Arteriosclerosis | 643 | 0 | 0.00% |
| Metabolic Dysregulation Syndrome | 643 | 0 | 0.00% |
| Hypertension | 643 | 0 | 0.00% |
| Diabetes | 643 | 0 | 0.00% |
| Electrolyte imbalances | 643 | 0 | 0.00% |
| Acute illness | 643 | 0 | 0.00% |
| Hyperlipidemia | 643 | 0 | 0.00% |
| Liver diseases | 643 | 0 | 0.00% |
| Hyperuricemia | 643 | 0 | 0.00% |
| Infectious diseases | 643 | 0 | 0.00% |
| Surgery within the past month | 643 | 0 | 0.00% |
| Active cancer | 643 | 0 | 0.00% |
| Lung infections | 643 | 0 | 0.00% |
| Recurrent nephrotic syndrome | 630 | 13 | 2.02% |
| Antiplatelet drugs | 643 | 0 | 0.00% |
| Diuretics | 643 | 0 | 0.00% |
| Chinese patent medicine for blood circulation... | 643 | 0 | 0.00% |
| Statins | 643 | 0 | 0.00% |
| Immunosuppressants | 643 | 0 | 0.00% |
| Anticoagulants | 643 | 0 | 0.00% |
| History of cancer | 643 | 0 | 0.00% |
| History of VTE | 643 | 0 | 0.00% |
| Smoking history exceeding 10 years | 643 | 0 | 0.00% |
| Alcohol consumption history exceeding 10 years | 643 | 0 | 0.00% |
| PMN duration | 643 | 0 | 0.00% |
| BNP > 100pg/mL | 643 | 0 | 0.00% |
| Hematocrit | 642 | 1 | 0.16% |
| Haemoglobin | 643 | 0 | 0.00% |
| MCV | 642 | 1 | 0.16% |
| hs-CRP > 5mg/L | 643 | 0 | 0.00% |
| PLT | 643 | 0 | 0.00% |
| NE | 641 | 2 | 0.31% |
| NEUT% | 641 | 2 | 0.31% |
| WBC | 642 | 1 | 0.16% |
| EO% | 641 | 2 | 0.31% |
| A/G | 641 | 2 | 0.31% |
| AST/ALT | 641 | 2 | 0.31% |
| ALB | 643 | 0 | 0.00% |
| ALB/uALB | 634 | 9 | 1.40% |
| GLB | 641 | 2 | 0.31% |
| AST | 641 | 2 | 0.31% |
| ALP | 642 | 1 | 0.16% |
| CHE | 642 | 1 | 0.16% |
| Cl | 642 | 1 | 0.16% |
| Cr | 643 | 0 | 0.00% |
| Urea | 643 | 0 | 0.00% |
| TP | 643 | 0 | 0.00% |
| Urea/Cr | 643 | 0 | 0.00% |
| UA | 642 | 1 | 0.16% |
| eGFR | 642 | 1 | 0.16% |
| CK-MB activity | 614 | 29 | 4.51% |
| MYO > 140ng/mL | 643 | 0 | 0.00% |
| Lp(a) | 630 | 13 | 2.02% |
| LDL-C | 632 | 11 | 1.71% |
| Hcy | 626 | 17 | 2.64% |
| APTT | 640 | 3 | 0.47% |
| APTR | 635 | 8 | 1.24% |
| TT | 639 | 4 | 0.62% |
| AT III activity | 502 | 141 | 21.93% |
| DD | 641 | 2 | 0.31% |
| FDP > 5mg/L | 640 | 3 | 0.47% |
| FIB | 640 | 3 | 0.47% |
| PT | 641 | 2 | 0.31% |
| INR | 641 | 2 | 0.31% |
| CA125 > 35U/mL | 638 | 5 | 0.78% |
| CK 19 fragment | 535 | 108 | 16.80% |
| AFP | 628 | 15 | 2.33% |
| PCT > 0.05ng/mL | 642 | 1 | 0.16% |
| umALB | 627 | 16 | 2.49% |
| umALB/Ucr | 625 | 18 | 2.80% |
| Ucr | 625 | 18 | 2.80% |
| 24h urine volume | 600 | 43 | 6.69% |
| 24h urinary protein | 600 | 43 | 6.69% |
| Urinary total protein | 643 | 0 | 0.00% |
| aPLA2Rab | 522 | 121 | 18.82% |
| ESR | 611 | 32 | 4.98% |
| FOB | 640 | 3 | 0.47% |
| Padua score | 634 | 9 | 1.40% |

S Tab 8 Medical Reference Ranges for Discretized Features and Model-Incorporated Characteristics

| **Indicators** | **Reference Range** |
| --- | --- |
| BNP | 0.0--100.0pg/mL |
| hs-CRP | 0--5.00mg/L |
| umALB/Ucr | 0.0—30.0mg/g |
| ALB | 40.0--55.0g/L |
| CHE | 5.0--12.0KU/L |
| MYO | 0.0--140.1ng/mL |
| AT III activity | 79.4--112% |
| DD | 0.00--0.55mg/L FEU |
| FDP | 0.0--5.0mg/L |
| INR | 0.96--1.16 |
| CA125 | <35.0U/mL |
| PCT | 0--0.05ng/mL |
| aPLA2Rab | 0.00--14.00RU/mL |

S Tab 9 Summary of Composite Feature

| **Composite Feature** | **Specific Contents** |
| --- | --- |
| anticoagulants | Rivaroxaban, Heparin, Nadroparin, Enoxaparin, Warfarin, Dabigatran |
| antiplatelet drugs | Indobufen, Aspirin |
| statins | Atorvastatin, Fluvastatin, Pravastatin, Rosuvastatin, Simvastatin |
| diuretics | Torasemide, Furosemide, Spironolactone |
| immunosuppressants | Prednisolone acetate, Methylprednisolone, Hydroprednisone, Dexamethasone, Hydrocortisone, Cyclophosphamide, Tacrolimus, Tripterygium glycosides, Rituximab |
| Liver diseases | Hepatic cyst, Hepatic insufficiency, Fatty liver, Hepatic calcification, Liver cirrhosis, Drug-induced liver injury, Decompensated liver cirrhosis |
| Metabolic Dysregulation Syndrome | Diabetic ketoacidosis, Metabolic acidosis, Respiratory acidosis |
| Acute illness | Acute gastroenteritis, Acute bronchitis, Acute cystitis, Acute renal insufficiency, Acute renal tubular injury, Acute left heart failure, Acute anterior wall myocardial infarction, Acute pyelonephritis, Acute upper respiratory infection, Acute coronary syndrome, Acute periapical periodontitis, Acute appendicitis, Acute tonsillitis |
| Lung diseases | Bilateral pulmonary bullae, Pulmonary bullae, Pulmonary nodules, Right lung destruction, Emphysema, Right upper lobectomy, Chronic obstructive pulmonary disease, Resection of right upper lobe adenocarcinoma, Pulmonary interstitial fibrosis, Space-occupying lesion of the lung, Bilateral ground-glass nodules, Interstitial lung disease, |
| Infectious diseases | Urinary tract infection, Foot skin infection, Skin and soft tissue infection, Upper respiratory infection, Bronchial infection, Herpes virus infection, Novel coronavirus infection, Oral infection, Methicillin-resistant Staphylococcus aureus infection, |
